# Inverse Drexhage Effect in Epsilon‐Near‐Zero Substrates

**DOI:** 10.1002/nap2.70225

**Published:** 2026-08-02

**Authors:** Sandeep Kumar Chamoli, Mohamed ElKabbash

**Affiliations:** ^1^ Harvard Medical School & Massachusetts General Hospital Charlestown Massachusetts USA; ^2^ James C. Wyant College of Optical Sciences University of Arizona Tucson Arizona USA

## Abstract

The Drexhage effect, caused by the interference between a dipole and its image formed in a substrate, modifies the local density of optical states of quantum emitters which can either enhance or suppress their spontaneous emission rate depending on the dipole orientation and distance from the substrate. Here, we show that for an epsilon‐near‐zero (ENZ) substrate, the observed orientation and distance dependence of the spontaneous emission rate is reversed compared to metals. This *inverse Drexhage effect* is studied for ideal ENZ and real ENZ substrates compared with ideal and real metallic substrates. ENZ metamaterials consisting of a subwavelength metal‐dielectric stack are shown to exhibit the conventional Drexhage effects due to the large optical losses associated with these materials. Our results could find applications in quantum sensing, quantum information, and energy‐efficient optoelectronic devices.

## Graphical abstract

Radiative emission of dipoles near substrates is shown to reverse between (a) perfect electric conductors and (b) epsilon‐near‐zero (ENZ) media. The schematic highlights switch from vertical to horizontal dipole dominance, governed by interference with image dipoles, leading to directional emission into the upper half‐space and enabling new strategies for controlling light–matter interactions.

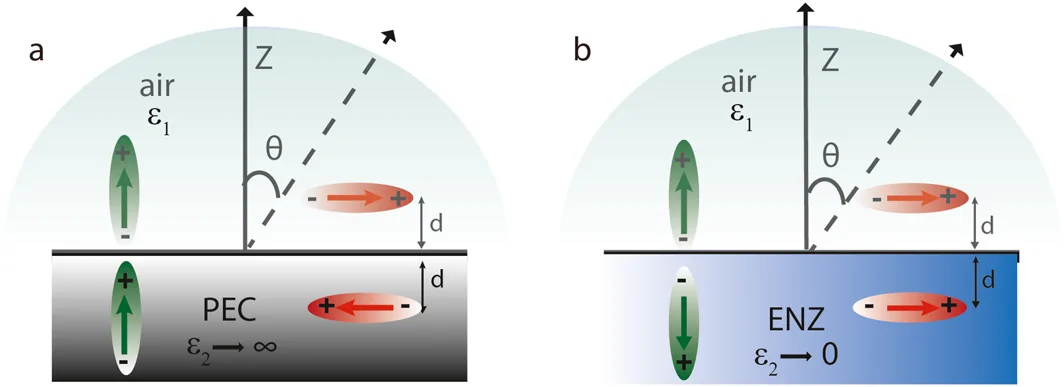

## 1 Introduction

The spontaneous emission (SE) rate is not an inherent property of a given emitter as it strongly depends on the surrounding electromagnetic environment. The emission rate is given by Fermi's golden rule [1].

(1) $$\Gamma_{ij}\;\propto \;{\left|M_{ij}\right|}^{2}\rho \left(\upsilon_{ij}\right),$$

where $\Gamma_{ij}$ is the rate for the transition between the excited state i and lower‐energy state j; $M_{ij}$, is a matrix element that connects the excited and lower energy levels and is determined by the wavefunctions associated with those levels and $\rho \left(\upsilon_{ij}\right)$ is the density of the optical field at the transition frequency (local density of optical states) [1]. Accordingly, the SE rate of an emitter is proportional to the number of propagating or evanescent electromagnetic modes available to the emitter to radiate into. The Purcell factor (PF) quantifies the ratio of the modified radiative decay rate to that in free space ΓgΓ0, where $\Gamma_{g}$ is the modified SE rate and $\Gamma_{0}$ is the SE rate in free space for an emitter [2]. The spontaneous emission rate of an emitter separated by a distance d from a mirror periodically oscillates between PF < 1 (rate suppression) and PF > 1 (rate enhancement) as a function of d with the overall modification of the emission rate dropping at large d [3]. This behavior, known as the Drexhage effect, represented one of the earliest quantum electrodynamics experiments. The Drexhage effect arises due to the distance dependent interference between the field radiated by the dipole and its image forming on the mirror substrate. This effect is widely used to probe the local density of optical states and to engineer radiative properties of emitters for applications in quantum optics, light‐emitting devices, and cavity QED [4, 5]. It provides a simple, geometry‐based approach to enhance or suppress emission without the need for nanostructured resonators. Recent studies have also explored emission control using structured ENZ systems, including ENZ nanoparticles [6], anisotropic multilayers [7], and nanorod–ENZ film hybrids [8]. Moreover, gradient ENZ stacks have been employed to achieve broadband thermal emission beaming [9, 10, 11]. The emission rate strongly depends on the emitter dipole's orientation; A charge q polarizes the surface of its substrate and induces an image charge $q^{\prime }=-S\;q$, where S is a complex amplitude given by the image coefficient $S=\left(\varepsilon_{2}-\varepsilon_{1}\right)/\left(\varepsilon_{2}+\varepsilon_{1}\right).$ For a perfect electrical conductor (PEC) or a dielectric substrate, the dipole and its image are in an opposite‐charge configuration (Figure 1a) [12, 13]. Accordingly, for horizontally (vertically) oriented dipoles, the image dipole is out‐of‐phase (in‐phase) and at distances *d* ≪λ, this results in PF<1(PF>1). Note, however, that if the emitter is near the metal substrate (d<20nm) the PF increases significantly, regardless of the dipole orientation, due to the excitation of surface plasmon polaritons and lossy surface waves. The high PF here, however, is due to the increase in the nonradiative component $F_{\text{non}-\text{rad}}$ rather than the radiative component $F_{\text{rad}}$ and does not improve the external quantum efficiency η of the emitter defined as:

(2) $$\eta =\frac{F_{\text{rad}}}{\left(F_{\text{rad}}+F_{\text{non}-\text{rad}}\right)}.$$

**FIGURE 1:**
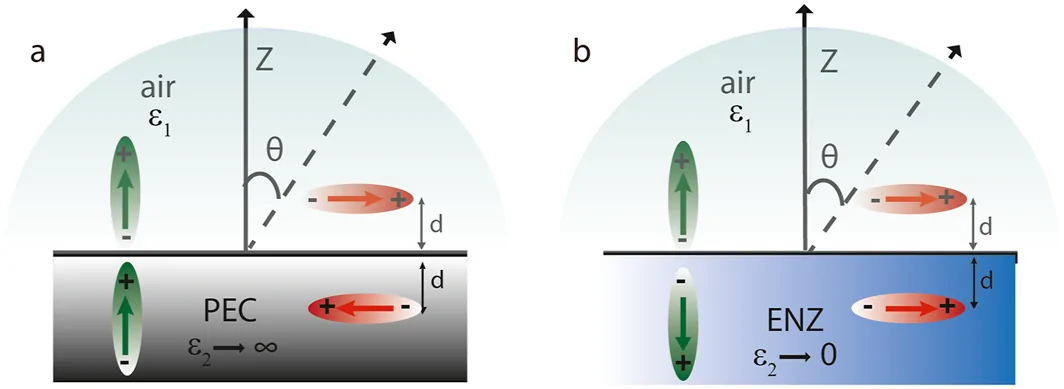
A schematic of the system under study. Horizontal and vertical dipole and its image dipole on (a) PEC ($\varepsilon_{2}\to \infty$) and (b) ENZ ($\varepsilon_{2}\to 0$) substrate. In the case of PEC, the vertical dipole interacts constructively with its image dipole, leading to an enhanced dipole moment, whereas the horizontal dipole is canceled by its image dipole. In contrast, for ENZ, the horizontal dipole undergoes constructive interference with its image dipole, resulting in an increased dipole moment, whereas the vertical dipole is canceled by its image dipole. The dipole is placed in air ($\varepsilon_{1}=1$). The radiative emission refers exclusively to power emitted into the upper (air) half‐space, corresponding to collection angles *θ* between 0° to 90°; since this determines the portion that can be collected experimentally.

Inversion of the conventional Drexhage orientation dependence has previously been reported in the context of magnetic mirrors [14], where transverse‐electric dipoles experience enhanced emission near reflectors exhibiting near‐zero reflection phase. Such magnetic‐mirror behavior can be realized at optical frequencies using high‐index dielectric nanostructures that emulate a magnetic response through resonant Mie modes [15]. In contrast, epsilon‐and‐mu‐near‐zero media have been studied primarily in bounded bulk configurations, where modified radiation characteristics and spontaneous emission control arise from near‐zero refractive index and uniform phase distributions within the medium [16]. In this work, we show that a simple planar ENZ exhibits the opposite response to a dipole, embedded in air/vacuum, interacting with a metallic or a dielectric substrate similar to magnetic mirrors, but without nanostructuring. This observation is due to the image coefficient obtained when $Re\;\left(\varepsilon_{subs}\;∼0\right)$ leading to S<0. Consequently, the dipole and its image are in a like‐charge configuration (Figure 1b) [13]. A direct consequence of this “Inverse Drexhage effect” is that for d≪λ, a horizontal dipole exhibits an increase in its emission rate and quantum efficiency when it is directly placed on an ENZ substrate. We show that ENZ Metamaterials, consisting of a multilayer stack of metal‐dielectric thin‐films, exhibit both the Drexhage and Inverse Drexhage effects depending on the dipole orientation [17]. We quantify how the complex permittivity of the ENZ substrate determines the transition between radiative enhancement and nonradiative quenching. The proposed effect is a promising and lithographically‐free approach to control the emission rate and quantum efficiency of quantum emitters with anisotropic dipole orientation such as two‐dimensional luminescent materials for quantum sensing of the local density of optical states, quantum information, and energy‐efficient optoelectronic devices [18, 19, 20, 21, 22, 23].

## 2 Analytical Model

An excited emitter in free space decays to its ground state according to the Larmor formula [1, 24].

(3) $$\Gamma_{0}=\frac{{\left|\mu \right|}^{2}\sqrt{\varepsilon }\omega^{3}}{3hc^{3}},$$

where μ is the electric dipole moment; ω is emission angular frequency; and ε is the permittivity of the host material. The PF of a dipole placed a distance d above a substrate with a horizontal (∥, “P‐polarization”) and vertical (⊥, “S‐polarization”) orientation is given by [1, 24]:

(4) $$PF_{⊥}=1-\eta_{0}\left(1-\frac{3}{2}\text{Re}\int_{0}^{\infty }dk_{x}\frac{1}{k_{z}}{\left(\frac{k_{x}}{\sqrt{\varepsilon_{1}}k_{0}}\right)}^{3}\left(1+r_{P}e^{2ik_{z}d}\right)\right),$$

(5) $${\text{PF}}_{∥}=1-\eta_{0}\left(1-\frac{3}{4}\text{Re}\int_{0}^{\infty }dk_{x}\frac{1}{k_{z}}\frac{k_{x}}{\sqrt{\varepsilon_{1}}k_{0}}\left[1+r_{S}e^{2ik_{z}d}+{\left(\frac{k_{z}}{\sqrt{\varepsilon_{1}}k_{0}}\right)}^{2}\left(1-r_{P}e^{2ik_{z}d}\right)\right]\right),$$

where $\eta_{0}$ is the internal quantum efficiency of the dipole in free space which is close to unity for R6G [25] and real permittivity $\varepsilon_{1}$, $k_{0}=\frac{2\pi }{\lambda_{0}}$ is the magnitude of the wavevector in vacuum at wavelength $\lambda_{0}$, $k_{z}=\sqrt{\varepsilon_{1}k_{0}^{2}-k_{∥}^{2}}$ and $k_{∥}=\sqrt{k_{x}^{2}+k_{y}^{2}}$, are the wavevector components along the vertical and horizontal or in‐plane directions, respectively, and $r_{P}$ and $r_{S}$ represent the amplitude reflection coefficients of the substrate for a P‐ (TM) or S‐ (TE) polarized plane wave and can be calculated using the [26]. The normalized dissipated power spectra are represented by the integrands in Equations (4) and (5), respectively.

(6) $$\frac{{dP}_{⊥}}{{dk}_{x}}=\frac{3}{2}\frac{1}{k_{z}}{\left(\frac{k_{x}}{\sqrt{\varepsilon_{1}}k_{0}}\right)}^{3}\left(1+r_{P}e^{2ik_{z}d}\right),$$

(7) $$\frac{{dP}_{∥}}{{dk}_{x}}=\frac{3}{4}\frac{1}{k_{z}}\frac{k_{x}}{\sqrt{\varepsilon_{1}}k_{0}}\left\{1+r_{S}e^{2ik_{z}d}+\frac{k_{z}^{2}}{\varepsilon_{1}k_{0}^{2}}\left[1-r_{P}e^{2ik_{z}d}\right]\right\}.$$

The dissipated power refers to the total power emitted by a dipole source into its surrounding environment, including both radiative (propagating) and nonradiative contributions. The nonradiative component comprises evanescent (high‐*k*) modes, which exist even in lossless systems, whereas material losses determine whether these channels lead to energy dissipation. The propagating‐wave contribution to spontaneous emission enhancement is obtained by integrating the normalized dissipated power over the wavevector range $0<k_{x}<k_{cf}$, where $k_{cf}=\sqrt{\varepsilon_{1}}k_{0}$ is the cutoff wavevector of the dielectric medium. In this range, only modes that satisfy $k_{x}<k_{cf}$ can escape into the far field at a corresponding emission angle. For $k_{x}>k_{cf}$, the emitted power includes evanescent, nonradiative components such as plasmonic modes and lossy surface waves, which remain confined near the surface [1]. Radiative enhancement refers to the ratio between the radiative emission from dipole emitters located on substrates to that from dipole emitters located in free space [25]

(8) $$F_{\text{rad},⊥/∥}=\text{Re}\frac{\int_{0}^{k_{cf}}dk_{x}\frac{dP_{⊥/∥}}{dk_{x}}}{\int_{0}^{k_{cf}}dk_{x}\frac{dP_{0}}{dk_{x}}},$$

where $P_{0}$ is the normalized dissipated power for emitters in free space.

## 3 Results and Discussions

We calculated the PF and $F_{\text{rad}}$ using Equations defined in theoretical model and compared the results with those obtained using the stackpurcell solver in the commercial software Lumerical, which is based on a planar Green‐function formalism. An electric point dipole was placed at a distance *d* from the substrate to model the interaction between the emitter and the structure. The $F_{rad}$ in FDTD was calculated by integrating the far‐field power density over emission angles *θ* ranging from 0° to 90°, corresponding to the propagating modes, $\left(k_{x}/k_{0}\leq 1\right)$ thereby excluding contributions from evanescent or nonradiating modes (see Figure 1). Figure 2a shows the calculated PF for dipoles placed above a perfect electrical conductor (PEC) substrate, where the conventional Drexhage effect is observed: at d≪λ, the emission rate is enhanced for the vertical dipole and suppressed for the horizontal dipole due to the image dipole effect [1]. Figure 2b shows the calculated Purcell factor for dipoles above an ideal, lossless, ENZ substrate, where the inverse Drexhage effect is clearly observed at d≪λ, the emission rate is enhanced for the horizontal dipole and suppressed for the vertical dipole. The results obtained from the analytical model based on Equations ((4), (5), (6), (7)) are compared with those calculated using the stackpurcell solver in Lumerical FDTD, which implements a multilayer Green's function approach, showing good agreement (see Supporting Information S1: Figure S1). In these calculations, the PEC is approximated using a material with a large complex refractive index, reproducing near‐unity reflection with a π‐phase shift, while remaining an approximation to the ideal boundary.

**FIGURE 2:**
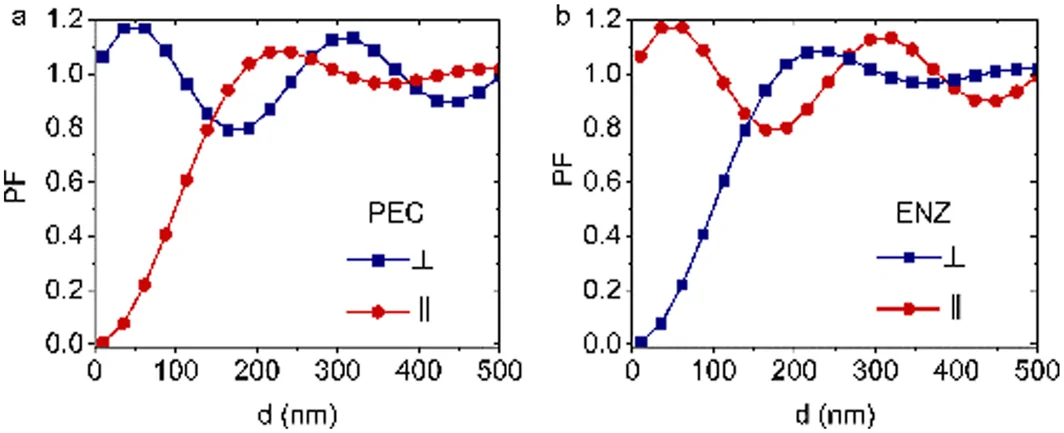
PF for vertical and horizontal in case of lossless (a) PEC substrate showing the conventional Drexhage effect and (b) ENZ substrate showing the inverse Drexhage effect.

In the idealized PEC and lossless ENZ systems, propagating transmission into the substrate is suppressed, such that $F_{rad}$ accounts only for power emitted into propagating modes in the upper half‐space ($k_{x}<k_{cf}$). Contributions with $k_{x}>k_{cf}$ correspond to evanescent (nonpropagating) modes, which remain present even in the absence of material loss, although they do not contribute to dissipative decay. Therefore, PF is equal to $F_{\text{rad}}$ only in the limit where the contribution from evanescent modes is negligible or absent, such that the total decay rate is dominated entirely by propagating radiative channels.

The $F_{\text{rad}}$ is calculated by integrating the far field power over 0–90 deg (see Supporting Information S1: Figures S2 and S3) or wave vectors ($k_{x}<k_{cf}$) at a given distance from the PEC and ENZ substrate (see Equation 8), represents a collection‐limited far‐field emission metric determined by the chosen angular (NA) integration range. In addition, the periodic oscillations of the emission rate is observed due to change in phase as a function of *d* [1].

For ENZ materials, the reflection phase for p‐polarized light increases smoothly from 0 to π with angle, whereas the s‐polarized phase stays near 0. For PEC, both phases remain π across all angles (see Supporting Information S1: Figure S4). These phase calculations are used to rederive Equations (9) and (10), which are used for PF calculation and match well with the results obtained from Equations (4) and (5) (see results in Supporting Information S1: Figure S5).

(9) $$\text{PF}⊥=\left(\frac{3}{2}\right)\int_{0}^{1}du\;\left(\frac{u^{3}}{w}\right)\left(1-\cos\left(x\;w\right)\right),$$

(10) $$\text{PF}\;∥=\left(\frac{3}{4}\right)\text{Re}\;\int_{0}^{1}du\;\left[\;\left(\frac{u}{w}\right)\left(1+e^{\left\{i\;x\;w\right\}}\;e^{\left\{-2i\;\text{si}n^{-1}\left(u\right)\right\}}\right)+u\;w\;\left(1+e^{\left\{i\;x\;w\right\}}\right)\right].$$

We now consider the effect of introducing losses to the ENZ substrate, that is, im(ε)≠0 in Figure 3. For both dipole orientations, introducing losses leads to a sharp increase in the PF (see Figure 3a,b), which, according to the definition in Equation (7), does not distinguish between radiative and nonradiative contributions to the emission rate. This increase is significant for d<20 nm which confirms the nonradiative (high‐*k*) origin of the high PF where the increase in the decay rate is associated with an increase in the local density of optical states due to the excitation of surface waves, and other dark modes in the substrate. The $F_{rad}$, on the other hand, decreases for horizontal dipoles due to the existence of nonradiative channels for the emitter (see Figure 3d). $F_{\text{rad}}$ > 1 is still obtained for im(ε)<1. On the other hand, $F_{\text{rad}}$ << 1 for the vertical dipole at all values for im(ε) (see Figure 3c). The increase in $F_{\text{rad}}$ at higher im(ε) for the vertical dipole is because the ENZ behavior no longer persists for high im(ε) and we obtain the conventional Drexhage effect [12, 13]. We also derive the dependence of image change $q^{\prime }$ on the imaginary part of the substrate, showing that for high imaginary permittivity, the sign flips (see Supporting Information S1: Note 1 and Equation 1). We have also discussed how the reflection phase varies as a function of the imaginary part of the ENZ material and the incidence angle in Supporting Information S1: Figure S6. In addition, we analyze the corresponding impedance mismatch at the air–substrate interface, which further explains the observed reflection phase behavior (see Supporting Information S1: Note 3 and Figure S7). A complementary sweep of the real part of the permittivity around the ENZ condition (Supporting Information S1: Figure S15a) confirms that the inverse Drexhage effect is confined to a finite spectral window centered at Re (ε) ≈ 0.

**FIGURE 3:**
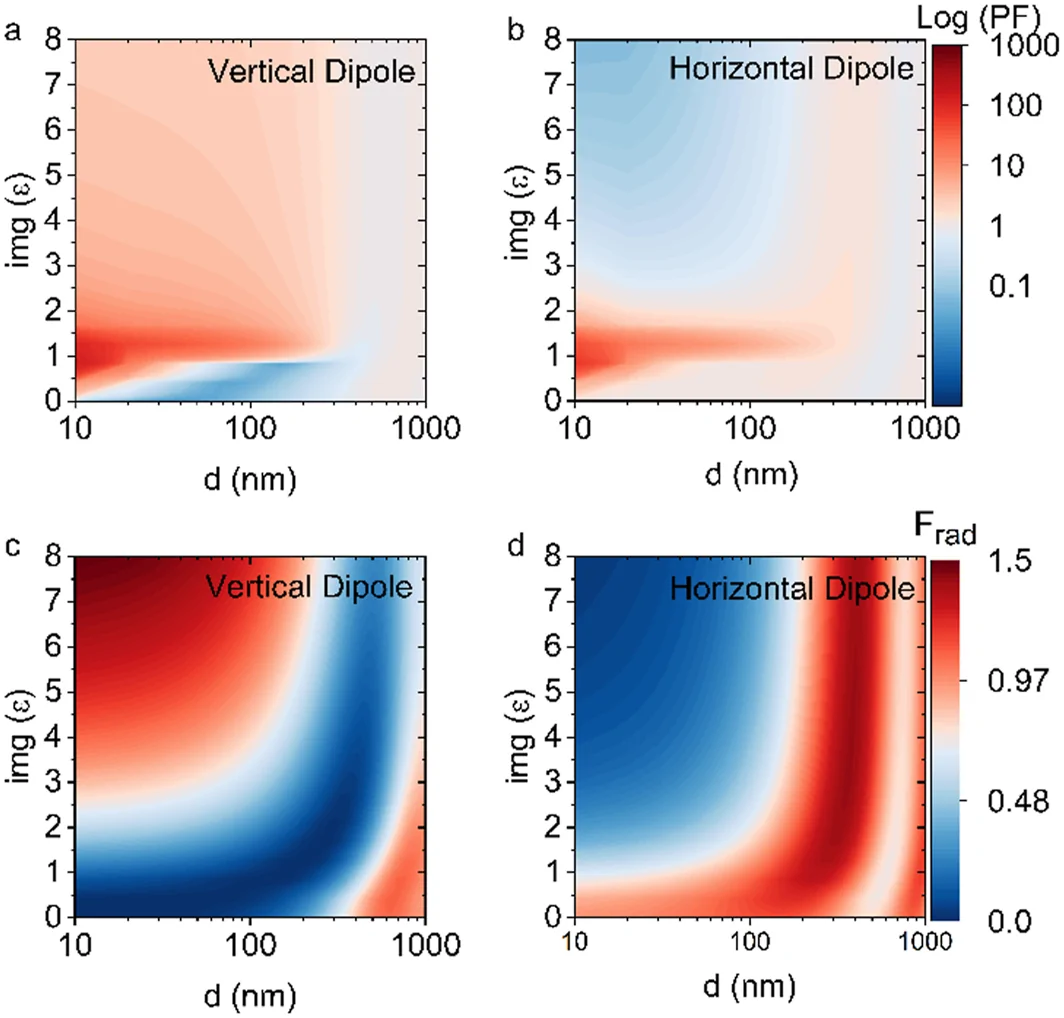
PF (logarithmic scale) and $F_{\text{rad}}$ for Horizontal dipole and Vertical dipole as a function of dipole distance d (logarithmic scale) and losses in the ENZ substrate keeping Re(ε)=0.01 at λ=1324nm. (a) PF for vertical dipole, (b) PF for horizontal dipole, (c) $F_{\text{rad}}$ for vertical dipole and (d) $F_{\text{rad}}$ for horizontal dipole.

Next, we compare the PF and $F_{\text{rad}}$ as a function of the emitter's distance from the substrate for several substrates—(a) a multilayer (ML) ENZ metamaterial “ENZ‐MM (ML),” (b) the same ENZ metamaterial with its permittivity calculated using the effective medium theory “ENZ‐MM (EM),” (c) Indium Tin Oxide (ITO), which is a lossy ENZ material at that wavelength, and (d) Silver (Ag). The effective permittivity of an ENZ metamaterial consists of a stack of alternating metal (TiN) [27]‐dielectric (TiO2) [28] layers shown in Figure 4a is calculated using the effective medium theory [17] (see Supporting Information S1: Note 2). Figure 4b shows the complex permittivity for ITO which is a conductive metal oxide [29].

**FIGURE 4:**
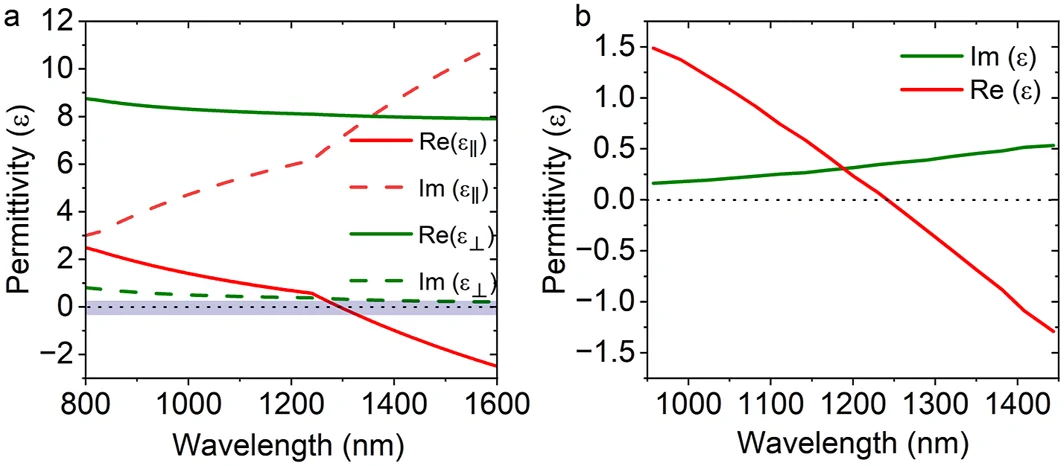
Permittivity of (a) ENZ MM (TiN (10 nm) + TiO2 (34 nm) and (b) ITO.

Figure 5 examines the PF and $F_{\text{rad}}$ at 1324 nm for substrates: 145 nm thick ITO, ENZ‐MM (ML) and 200 nm thick Ag. As shown in Figure 5a,b, both horizontal and vertical dipoles exhibit a significant PF enhancement for ENZ‐MM and ITO at short distances with respect to the wavelength (i.e., when d≪λ). This enhancement arises due to the direct coupling between the dipole field and the surface plasmon modes, ENZ modes, and Ferrel‐Berreman modes of ITO [30, 31], as well as the high‐κ bulk plasmon modes available in HMM [17, 25]. Ag shows relatively low PF due to the low losses at 1324 nm and exhibits a typical Drexhage behavior [25]. Figure 5c shows $F_{\text{rad}}$ for horizontal dipoles, where it is evident that lossy ITO substrates exhibit $F_{rad}>$ 1 at distances d≪λ around 200 nm, whereas the vertical dipole (Figure 5d) exhibits a significant drop in $F_{\text{rad}}$ demonstrating the inverse Drexhage behavior for horizontal dipoles. We also explored $F_{\text{rad}}$ and η for the ITO substrate as a function of dipole distance, ranging from near‐field to 500 nm, and analyze its behavior within and outside the ENZ wavelength range (see Supporting Information S1: Figure S8). Conversely the Ag substrate demonstrates the classical Drexhage effect for both the horizontal (Figure 5c) and vertical dipoles (Figure 5d). We also calculate the $F_{rad}$ behavior for Ag and ITO in case of isotropic dipole (see Supporting Information S1: Figure S9 and S10 respectively). The robustness of this behavior with respect to material parameters is further confirmed by varying the carrier concentration in ITO (Supporting Information S1: Figure S15b), where only modest changes in the emission rate are observed for ± 10% variation. The ENZ‐MM is an interesting case. As shown in Figure 4, these multilayer metamaterials exhibit an anisotropic permittivity with the parallel permittivity crossing epsilon zero region at ∼ 1310 nm. The behavior of the horizontal dipole is very similar to the Ag substrate because of the high im(ε) (Figure 4a), which makes the multilayer system, for the horizontal dipole, act as a highly lossy ENZ material which exhibits the conventional Drexhage effect (Figure 3d). On the other hand, the vertical dipole behaves similar to a dipole sitting on a dielectric substrate which also exhibits a conventional Drexhage effect (see Supporting Information S1: Figure S11). Additionally, the emission behavior remains qualitatively unchanged under variations of the metal fill fraction in the multilayer structure (Supporting Information S1: Figure S15c), indicating that the effect does not rely on fine‐tuned geometrical parameters. The behavior of a quantum emitter on the effective medium equivalent of the multilayer system, that is, ENZ MM (EM), is also modeled to ensure that the results are not due to the multilayer nature of the metamaterial (see Supporting Information S1: Figure S12). The validity of the effective medium approximation is quantitatively assessed in Supporting Information S1: Figure S15d, showing that it remains accurate (error < 10%) down to emitter–surface separations of ∼ 10–30 nm, depending on dipole orientation [32]. To explicitly verify the underlying energy balance and the validity of the ENZ approximation, we analyze the angular (*k*‐space) distribution of reflection, transmission, and absorption, as well as the spectral decomposition of the dissipated power (see Supporting Information S1: Figures S13 and S14). These results confirm that the approximation of near‐unity reflection and negligible transmission holds only in the ideal ENZ limit and within the propagating (low‐*k*) regime, whereas significant deviations arise in realistic lossy systems due to high‐*k* and absorptive channels.

**FIGURE 5:**
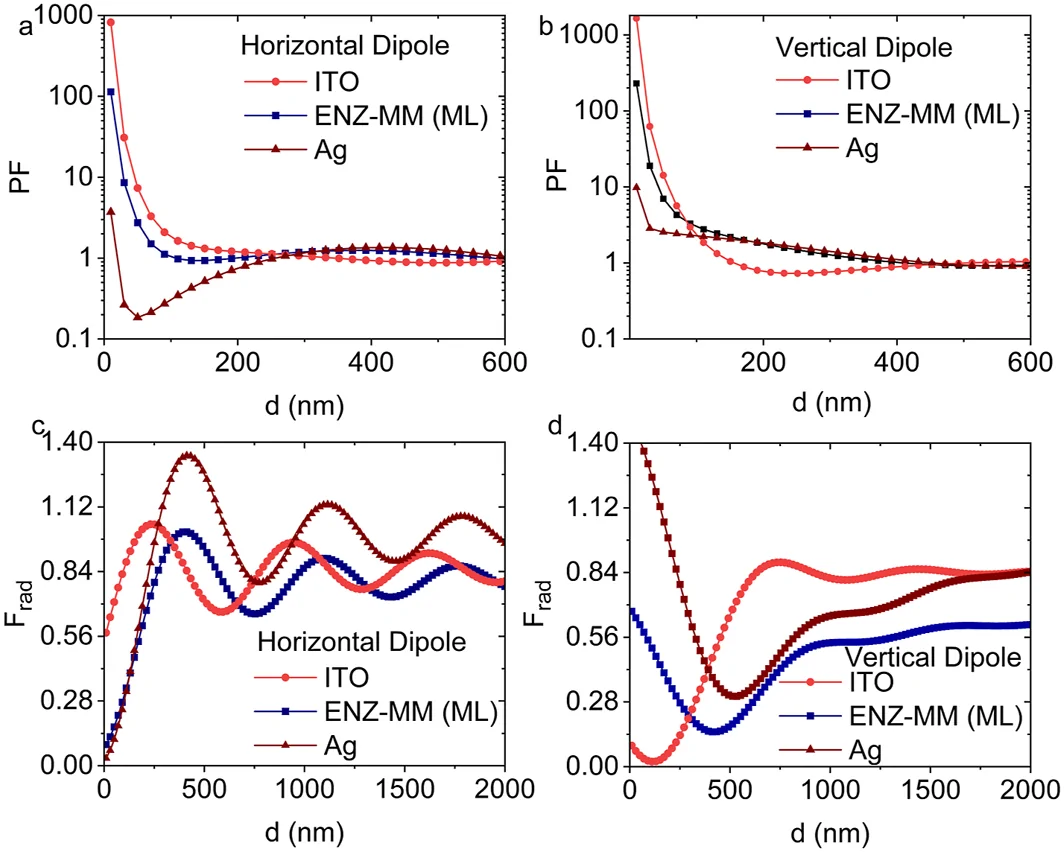
PF and $F_{\text{rad}}$ for horizontal and vertical dipole near ITO, ENZ MM using multilayer system (ML) and Ag substrate at 1324 nm. (a) PF for Horizontal dipole (b) PF for Vertical dipole, (c) $F_{\text{rad}}$ for horizontal dipole and (d) $F_{\text{rad}}$ for Vertical Dipole.

## 4 Conclusion

In conclusion, we have demonstrated that ENZ substrates reverse the orientation‐dependent behavior of spontaneous emission observed in conventional Drexhage experiments. We showed that horizontal dipoles exhibit enhanced radiative decay rates and extraction efficiencies when placed near ideal and real ENZ materials, such as ITO, whereas vertical dipoles are suppressed. In contrast, ENZ metamaterials represent an interesting class as they are highly anisotropic leading to the observation of rich dipole orientation dependence. In particular, the horizontal dipole orientation exhibited a conventional Drexhage behavior, even at ENZ wavelengths, due to the high optical losses of the ENZ metamaterial, whereas the metamaterial behaved as a dielectric substrate for vertical dipole orientations. The ability to enhance emission from horizontal dipoles—predominant in 2D materials such as transition metal dichalcogenides [4] using a planar, lithography‐free ENZ substrate offers a practical platform for improving the brightness and efficiency of quantum emitters at nanometric distances. This control can also be used for mapping the local density of optical states and for selectively suppressing or enhancing emission in optoelectronic devices, introducing the substrate type as a new degree of freedom for emission engineering [7, 8, 33] even without nanolithography. Similar effects may occur in Dirac‐cone ENZ media, though the angularly narrow near‐zero region may limit their utility for broadband spontaneous emission control [34]. Future work should focus on developing low‐loss ENZ metamaterials, where both horizontal and vertical dipoles could exhibit symmetric distance‐dependent emission, for example, if the imaginary permittivity of the ENZ metamaterial was below 1 as shown in Figure 3d. Such behavior would be of major importance for quantum emitters with isotropic orientation (due to symmetry), such as fluorophores embedded in dielectric matrices, by enhancing or suppressing the emission rate of both vertical and horizontal dipole orientations, which can expand the application of the Drexhage effect toward bioimaging, lighting, and quantum photonic integration.

## Author Contributions

**Sandeep Kumar Chamoli:** conceptualization, methodology, software, validation, investigation, formal analysis, visualization, writing – original draft, writing – review and editing. **Mohamed ElKabbash:** conceptualization, methodology, supervision, resources, writing – original draft, validation, investigation, writing – review and editing.

## Funding

The authors have nothing to report.

## Conflicts of Interest

The authors declare no conflicts of interest.

## Supporting information


Supporting Information S1

## Data Availability

Data are available upon request from the author.
