# Supplementary material for: Inverse Drexhage Effect in Epsilon‐Near‐Zero Substrates
Source: Nanophotonics. 2026 Aug 2;15(15):e70225. doi: 10.1002/nap2.70225 (PMC13642616; doi:10.1002/nap2.70225)
Supplement: Supplementary file 1 [file NAP2-15-e70225-s001.pdf]

Inverse Drexhage effect in Epsilon-Near-Zero Substrates

Sandeep Kumar Chamoli[1] and Mohamed ElKabbash[2]
[1)]*Harvard Medical School & Massachusetts General Hospital, Charlestown, MA 02129, USA*
[2)]*James C. Wyant College of Optical Sciences, University of Arizona, Tucson, Arizona 85721, USA*

(**Corresponding authors:** melkabbash@arizona.edu, chamolisandeep28@gmail.com)

(Dated: 4 July 2026)

Supplementary Figure S1

Purcell Factor (PF) comparison between simulation and theory for PEC and ENZ cases.

We have calculated the Purcell factor theoretically and compared it with simulation results. The theoretical expression employed is detailed in the manuscript, following the methodology outlined in [1]. Supplementary Figure S1 presents the comparison between theoretical and simulated results for both ENZ and PEC cases. For the ENZ case, the agreement is excellent. In contrast, for the PEC case, a noticeable difference arises due to the theoretical model using a simplified complex refractive index (real part = 700, imaginary part = 700), whereas the FDTD simulation implements a PEC model based on infinite conductivity. Despite this, the overall dipole behavior remains consistent in both approaches.

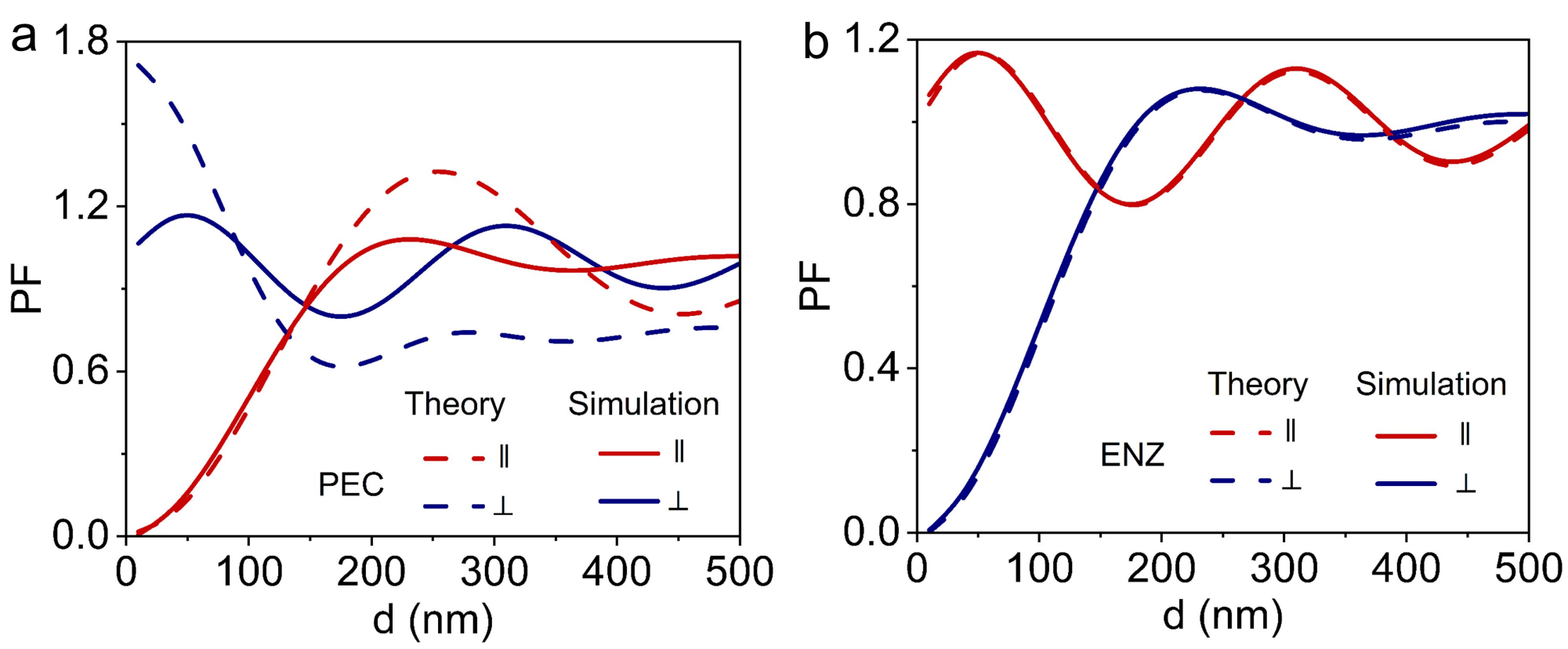

Figure S1: Comparison of the PF calculated theoretically and obtained from FDTD simulations for (a) the PEC case and (b) the ENZ case.

SUPPLEMENTARY NOTE 1: PERMITTIVITY CALCULATION FOR HYPERBOLIC METAMATERIAL (HMM)

The permittivity in Figure 4a for the parallel ($\varepsilon_{\parallel}$) and perpendicular ($\varepsilon_{\perp}$) components of the HMM is calculated using the following expressions[2]:

$$\varepsilon_{\parallel} = f_m\varepsilon_m + f_d\varepsilon_d \tag{1}$$

$$\varepsilon_{\perp} = \frac{\varepsilon_m\varepsilon_d}{f_m\varepsilon_m + f_d\varepsilon_d} \tag{2}$$

Here, ε_d and ε_m are the complex permittivities of TiO_2 and TiN, respectively. The fill fraction of TiN is given by $f_m = \frac{t_m}{t_m+t_d}$ and the fill fraction of TiO_2 is $f_d = 1 - f_m$, which depends on the thicknesses of TiN (t_m) and TiO_2 (t_d). The total thickness $t_m + t_d$ constitutes one unit cell.

Supplementary Figure 2: Radiative rate ($F_{\mathbf{rad}}$) for PEC and ENZ

Figure S2a presents the radiative rate (F_{rad}), showing the same trend as the Purcell factor (PF) for both dipole orientations, since PECs are lossless. Figure S2b presents the radiative rate for the ENZ configuration, following the same trend as the PF for both dipole orientations.

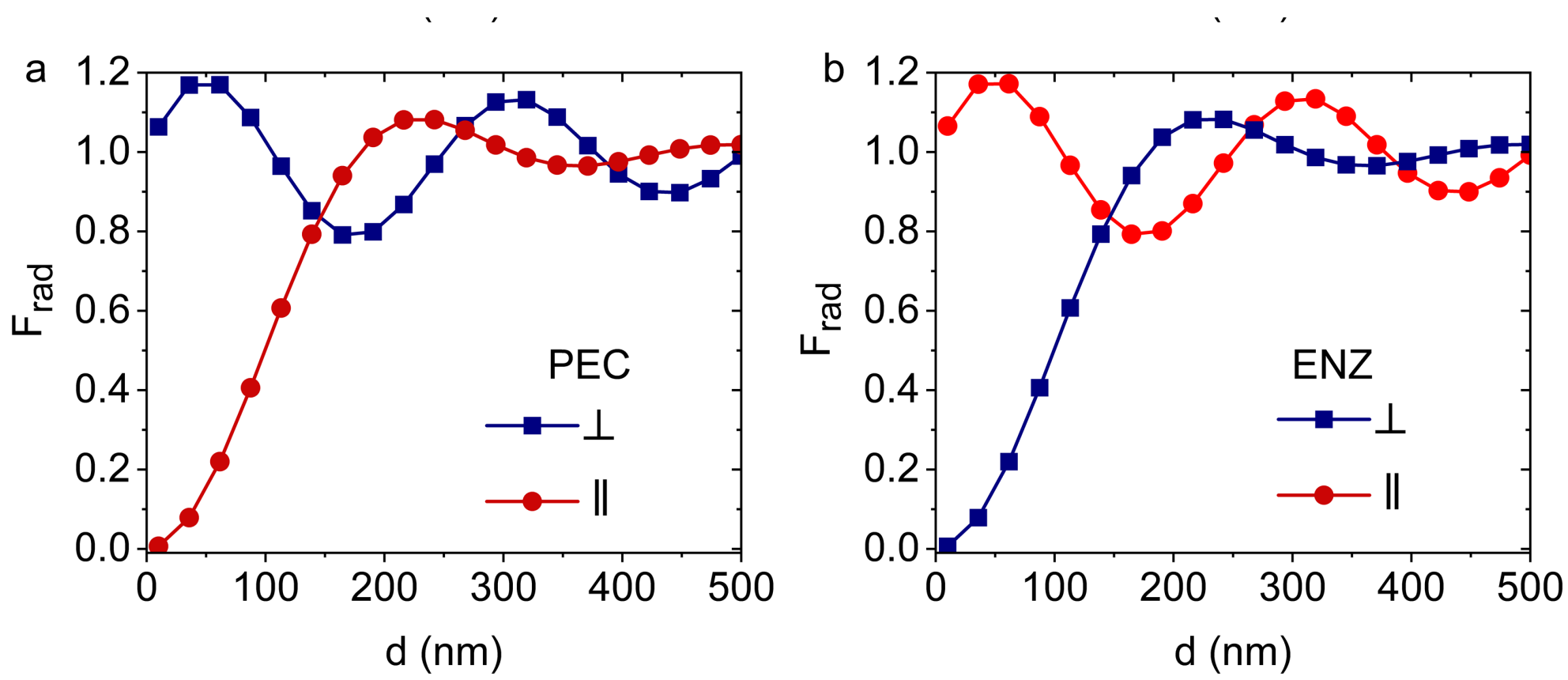

Figure S2: (a) F_{rad} for dipoles above a PEC substrate, showing the conventional Drexhage effect, and (b) F_{rad} for dipoles above an ENZ substrate, showing the inverse Drexhage effect.

Supplementary Figure 3: Far-field power density as a function of emission angle θ

Figure S3a and Figure S3b show the far-field power density as a function of emission angle θ and distance for horizontal and vertical dipoles. The results clearly illustrate the angle-dependent interference effect, with the dipole radiative energy varying sinusoidally.

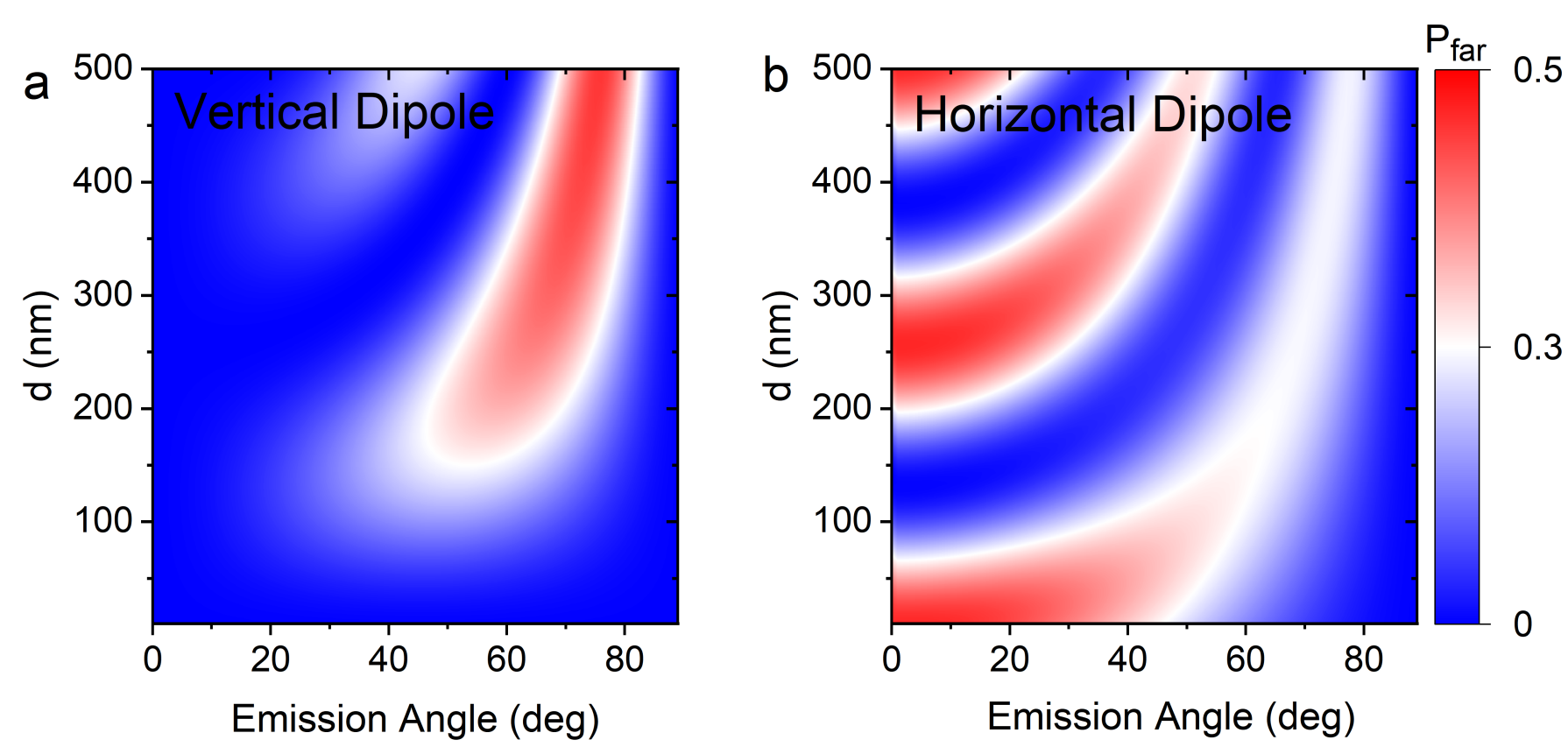

Figure S3: Power density per unit solid angle θ, as a function of far-field emission angle — (a) for vertical dipole and (b) for horizontal dipole — as a function of the collection angle and the inter-distance d between the dipole and the substrate.

Supplementary Figure 4: Reflection phase at the air–PEC and air–ENZ interface using Fresnel equations

For an interface between two media with refractive indices n_1 and n_2, the Fresnel reflection coefficients are:

$$r_s = \frac{n_1\cos\theta_1 - n_2\cos\theta_2}{n_1\cos\theta_1 + n_2\cos\theta_2}, \quad r_p = \frac{n_1\cos\theta_2 - n_2\cos\theta_1}{n_1\cos\theta_2 + n_2\cos\theta_1}$$

The reflection phase is given by:

$$\phi_s = \arg(r_s), \quad \phi_p = \arg(r_p)$$

For a perfect electric conductor (PEC), $n_2 \to \infty$, which leads to total reflection:

$$r_s = r_p = -1 \Rightarrow \phi_s = \phi_p = \pi$$

The reflection phase is constant (π) for all angles of incidence.

For an ENZ material, let $n_2 = \varepsilon + i\varepsilon$, where $\varepsilon \ll 1$. At normal incidence ($\theta_1 = 0$), the Fresnel reflection coefficients simplify to:

$$r_s \approx r_p \approx 1 - 2i\varepsilon \Rightarrow \phi_s = \phi_p \approx 0$$

As the angle of incidence increases, the TE (s-polarized) reflection phase ϕ_s remains close to zero, while the TM (p-polarized) reflection phase ϕ_p increases smoothly from 0 to π, as shown in Figure S4.

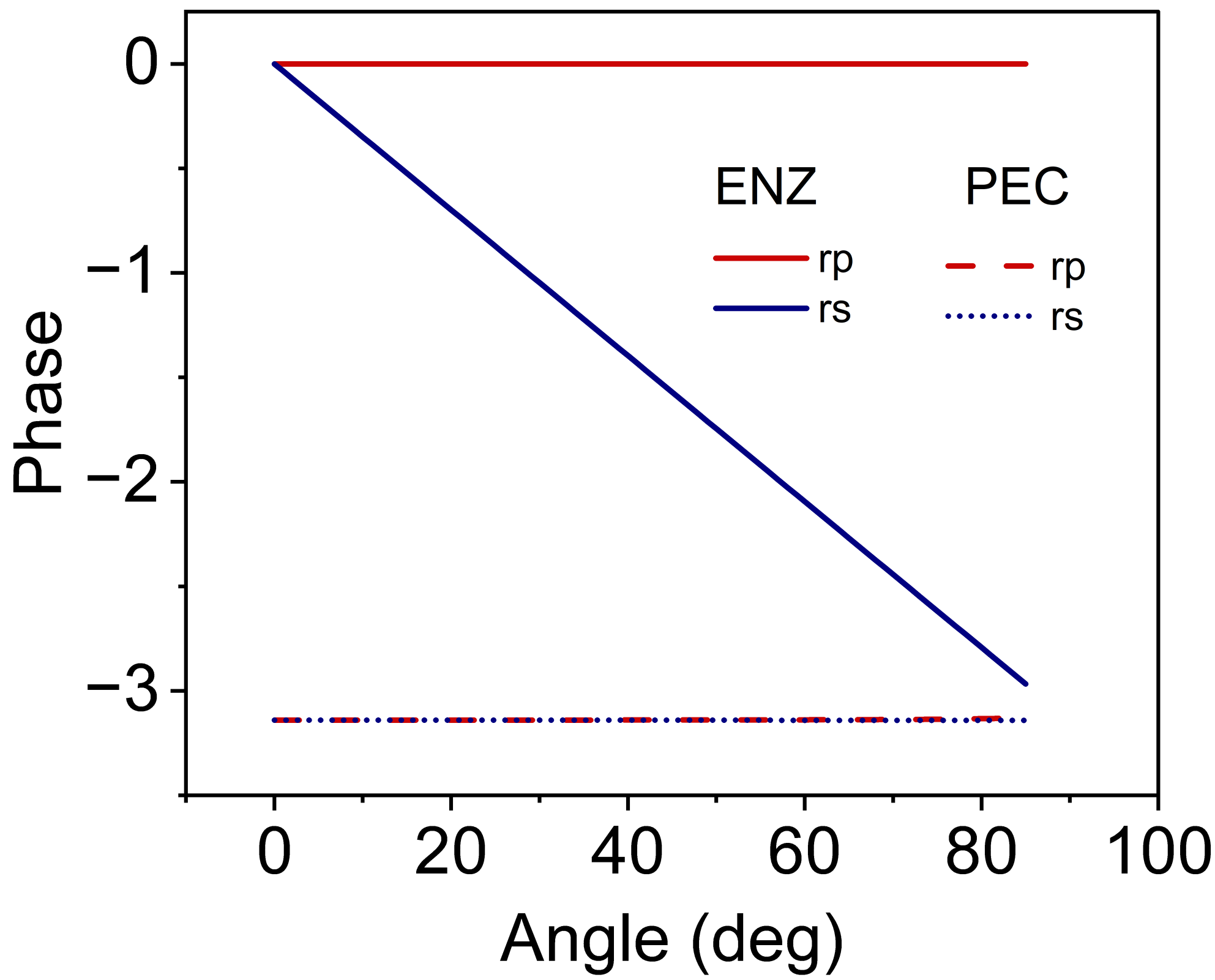

Figure S4: Reflection phase as a function of incident angle for ENZ ($700 + 700i$) and PEC ($n = 0.001$) using Fresnel equations.

Supplementary Figure 5: PF calculation for PEC and ENZ derived using Equations (4) and (5) from the main text

Now substituting $\eta_0 = 1$, $r_s \approx r_p \approx 1 - 2i\varepsilon$, and using the change of variable $u = \frac{k_x}{\sqrt{\varepsilon_1}k_0}$, then:

$$k_x = u\sqrt{\varepsilon_1}k_0, \quad dk_x = \sqrt{\varepsilon_1}k_0\,du, \quad k_z = \sqrt{\varepsilon_1}k_0 w, \quad \text{where } w = \sqrt{1-u^2}$$

Also let $x = 2k_0 d$. Then, Equations (4) and (5) in the main text for the ENZ case can be rewritten as:

$$\mathrm{PF}_\perp = \frac{3}{2}\int_0^1 du\,\frac{u^3}{w}\,(1 - \cos(xw)) \tag{3}$$

$$\mathrm{PF}_{\parallel} = \frac{3}{4}\,\mathrm{Re}\int_0^1 du\left[\frac{u}{w}\left(1+e^{ixw}e^{-2i\sin^{-1}(u)}\right)+uw\left(1+e^{ixw}\right)\right] \tag{4}$$

The PF plotted in Figure S5 using Equations (3) and (4) matches well with the theoretical PF values shown in Figure S1.

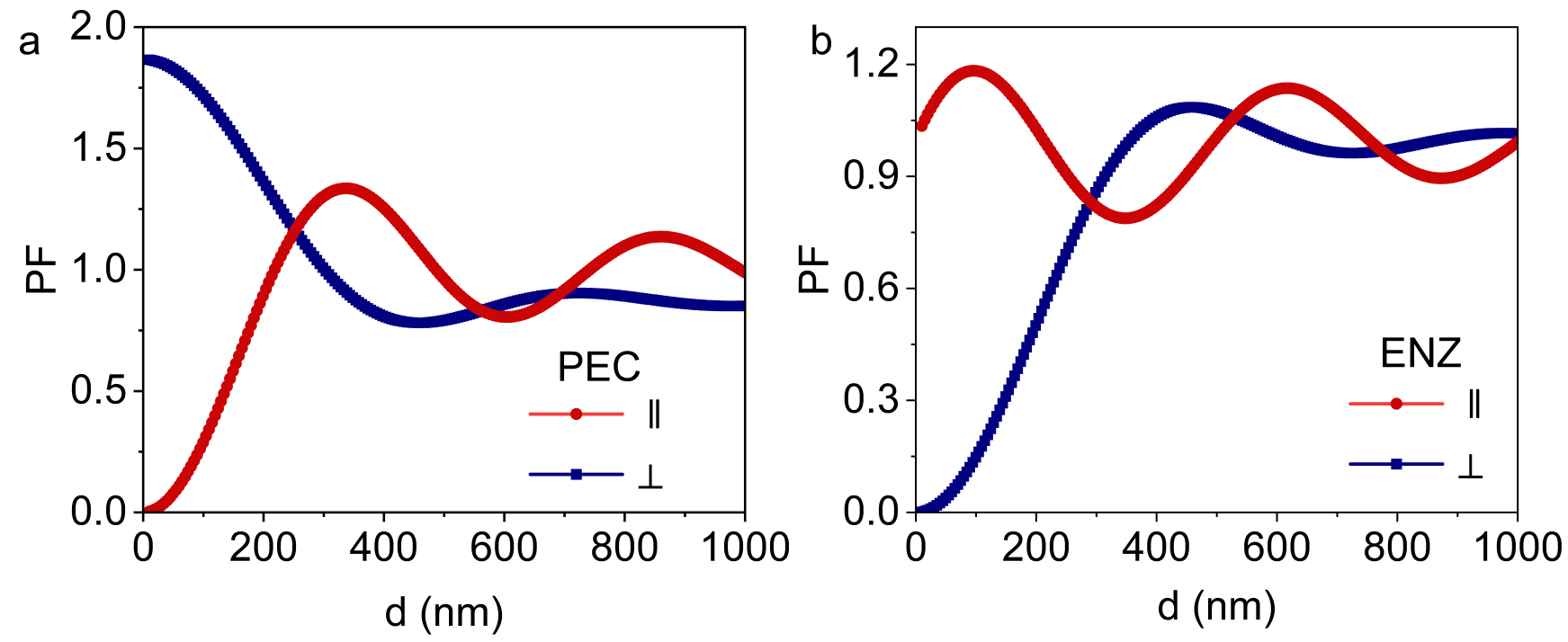

Figure S5: PF for PEC and ENZ substrates calculated using Equations (9) and (10) from the main text. These results show good agreement with the theoretical PF values presented in Figure S1, which were obtained using Equations (4) and (5).

Supplementary Note 2: Complex Image Charge

We can see the effect of the imaginary part of the substrate explicitly on the image charge modification using the following expression for q':

$$q' = q\cdot\frac{(\varepsilon_0^2+\mathrm{Re}[\varepsilon_{\mathrm{subs}}]^2+\mathrm{Im}[\varepsilon_{\mathrm{subs}}]^2)+i\,2\varepsilon_0\,\mathrm{Im}[\varepsilon_{\mathrm{subs}}]}{(\varepsilon_0+\mathrm{Im}[\varepsilon_{\mathrm{subs}}])^2+\mathrm{Im}[\varepsilon_{\mathrm{subs}}]^2} \tag{5}$$

Here, the presence of an imaginary component in the substrate permittivity alters both the magnitude and phase of q', leading to a complex image charge that affects field distribution and dipole interactions near the surface. As a result, the dipole image can shift and deform — especially as the dipole moves further from the surface — modifying its orientation and radiation pattern.

To clarify the transition from inverse to conventional Drexhage effects, we analyzed the reflection phase of *s*- and *p*-polarized waves as a function of the imaginary part of the ENZ refractive index (loss) and the incidence angle.

For *p*-polarized light (Figure S6b), the reflection phase remains close to 0° for all angles, even as the imaginary part of ENZ increases. This near-zero phase leads to constructive interference for the horizontal dipole, which explains the consistently higher Purcell factor for the parallel dipole near ENZ.

In contrast, for *s*-polarized light (Figure S6a), the phase is near 0° only at normal incidence, but gradually shifts toward −180° at larger angles or higher material losses. This transition reduces the constructive interference for the vertical dipole and makes it behave more like a conventional metal interface at higher losses.

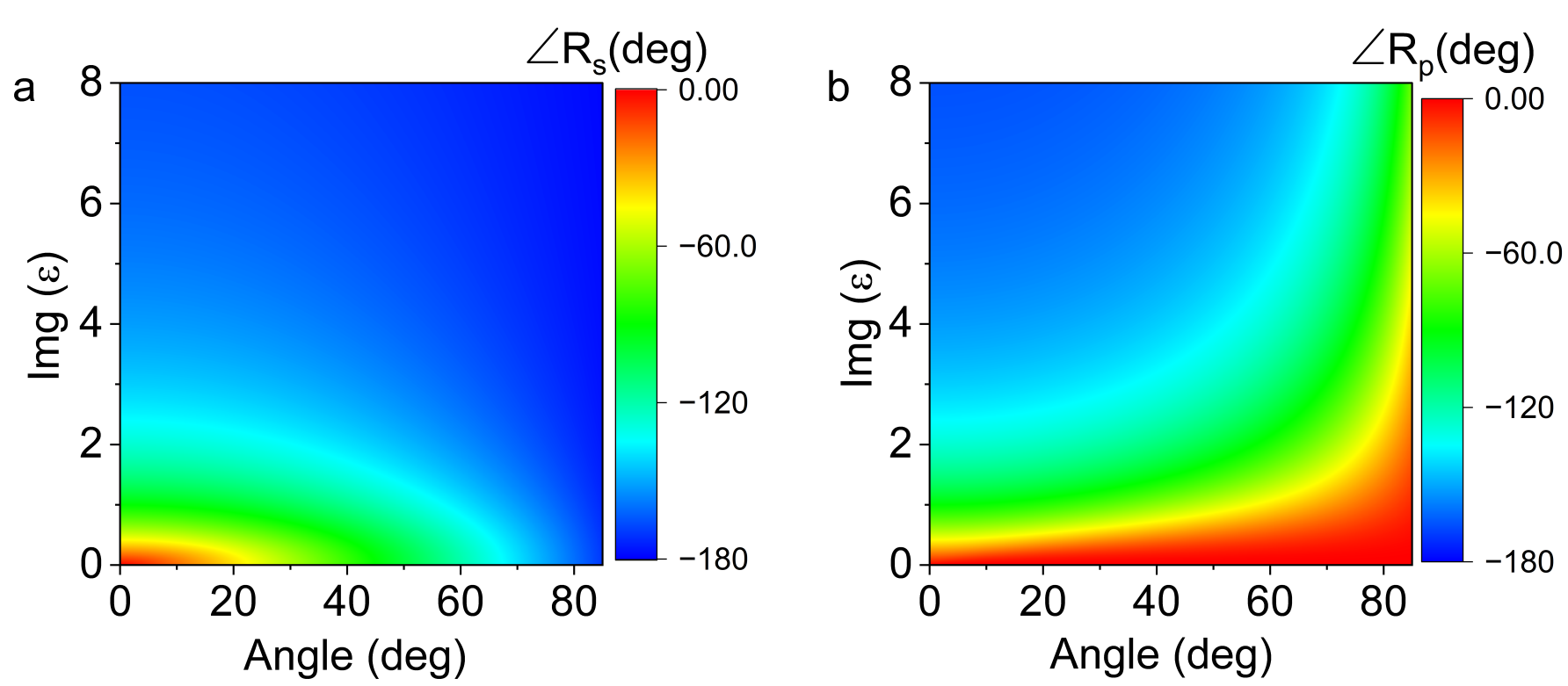

Figure S6: Reflection phase as a function of incidence angle and the imaginary part of ENZ: (a) for *s*-polarized light and (b) for *p*-polarized light.

Supplementary Note 3: Impedance at the Interface Between Two Media

For a plane wave in medium m, the intrinsic wave impedance is:

$$Z_m = \frac{\vec{E}}{\vec{H}} = \frac{Z_0}{n_m} \tag{6}$$

But when the wave is oblique, the impedance in medium m depends on polarization and angle:

$$Z_s = \frac{Z_0 \cos\theta_m}{n_m}, \quad Z_p = \frac{Z_0}{n_m \cos\theta_m} \tag{7}$$

So, inside the substrate medium 2:

$$Z_2 = \frac{Z_0 \cos\theta_2}{n_2}, \quad Z_p = \frac{Z_0}{n_2 \cos\theta_2} \tag{8}$$

Impedance to the ambient medium impedance (medium 1):

$$Z_1 = \frac{Z_0 \cos\theta_1}{n_1}, \quad Z_p = \frac{Z_0}{n_1 \cos\theta_1} \tag{9}$$

So, the relative impedance ratio between medium 2 and medium 1 is:

$$Z_s = \frac{\cos\theta_2}{n_2 \cos\theta_1}, \quad Z_p = \frac{\cos\theta_1}{n_2 \cos\theta_2} \tag{10}$$

Figure S7 shows the angular dependence of normalized wave impedance for ENZ and PEC substrates under both s- and p-polarized excitation. For the PEC case (Fig. S7b), the impedance remains near zero across all angles for both polarizations, consistent with the ideal behavior of a perfect electric conductor that enforces zero tangential electric field at the boundary. This near-zero impedance results in a strong impedance mismatch with air, leading to total reflection with a fixed phase shift of π.

In contrast, the ENZ case (Fig. S7a) displays a strongly angle- and polarization-dependent impedance. For p-polarized light, the impedance increases dramatically with angle, becoming extremely large at grazing incidence due to the field enhancement inside the ENZ medium. For s-polarized light, the impedance is extremely high at normal incidence but decreases rapidly with increasing angle. This distinct behavior stems from the reflection phase response shown in Fig. S4: while both polarizations experience a fixed π phase shift upon reflection from PEC, the ENZ substrate exhibits a polarization-dependent phase response.

Specifically, for p-polarized light, the reflection phase from ENZ remains near zero across all angles, indicating in-phase reflection. However, for s-polarized light, the reflection phase transitions gradually from 0 to π as the angle increases — a signature of the evolving impedance mismatch due to changes in the tangential electric field distribution at the interface. This phase evolution explains the angle-dependent impedance drop observed for s-polarized waves in ENZ.

The relationship between the reflection coefficient and impedance for s- and p-polarizations is given by expressions:

$$r_s = \frac{Z_{2,s} - Z_{1,s}}{Z_{2,s} + Z_{1,s}}, \quad r_p = \frac{Z_{2,p} - Z_{1,p}}{Z_{2,p} + Z_{1,p}} \tag{11}$$

For a PEC, $Z \to 0$, so at the air–PEC interface the impedance mismatch is extreme, leading to r_s and $r_p \to -1$ for all angles (perfect reflection with a π-phase shift). For ENZ at normal incidence, $Z \to \infty$, so at the air-ENZ interface, r_s and $r_p \to 0$.

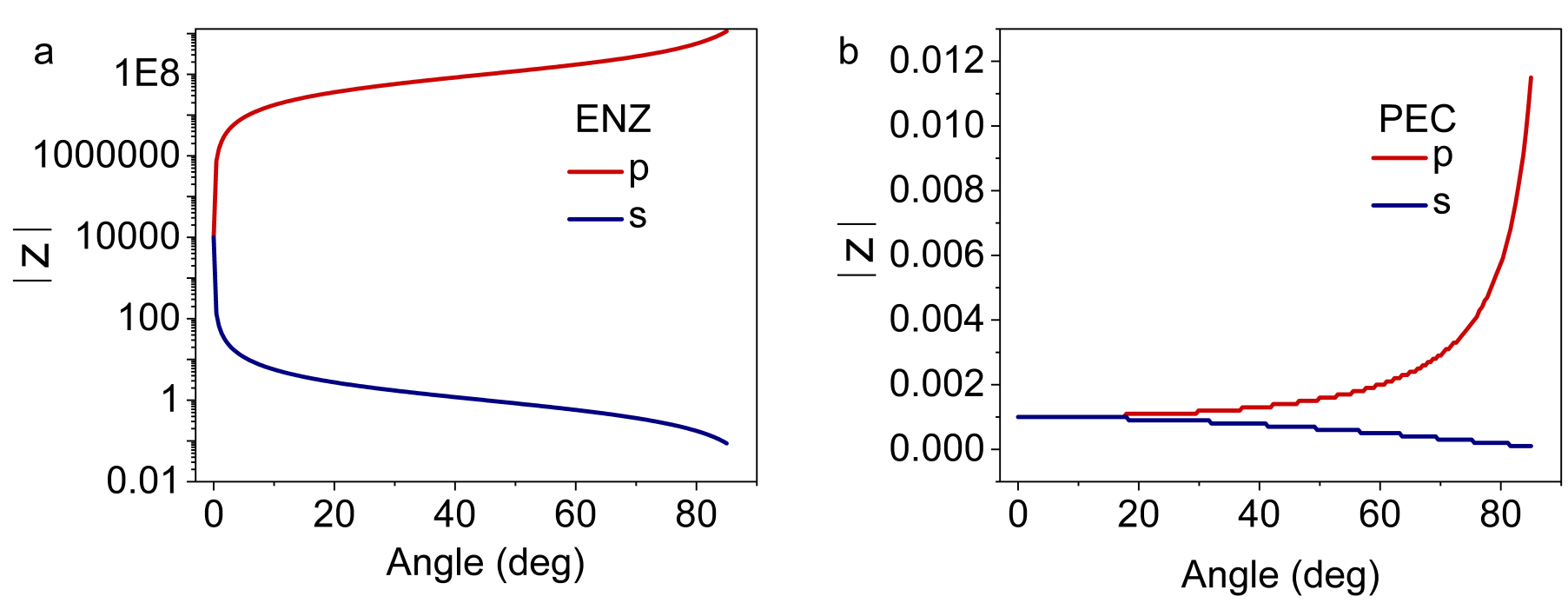

Figure S7: Impedance as a function of incident angle for (a) ENZ and (b) PEC.

SUPPLEMENTARY NOTE 4 AND SUPPLEMENTARY FIGURE S8: EXTRACTED QUANTUM EFFICIENCY

For the vertical dipole, Figure S3a shows the η_{ext}, while Figure S3c presents F_{rad} as the dipole moves from 10 nm to 500 nm across wavelengths from 900 nm to 1400 nm, covering both the ENZ region and pre-ENZ wavelengths of ITO. F_{rad} remains less than 1 almost everywhere across all wavelengths and distances, leading to very low η_{ext} for the vertical dipole, as expected due to the formation of an image dipole in the ITO that cancels out the vertical dipole's field, suppressing radiative decay and resulting in low extraction efficiency.

On the other hand, for the horizontal dipole, Figure S3b shows significantly higher η_{ext}, and $F_{\text{rad}} > 1$ in Figure S3d due to the inverse Drexhage effect.

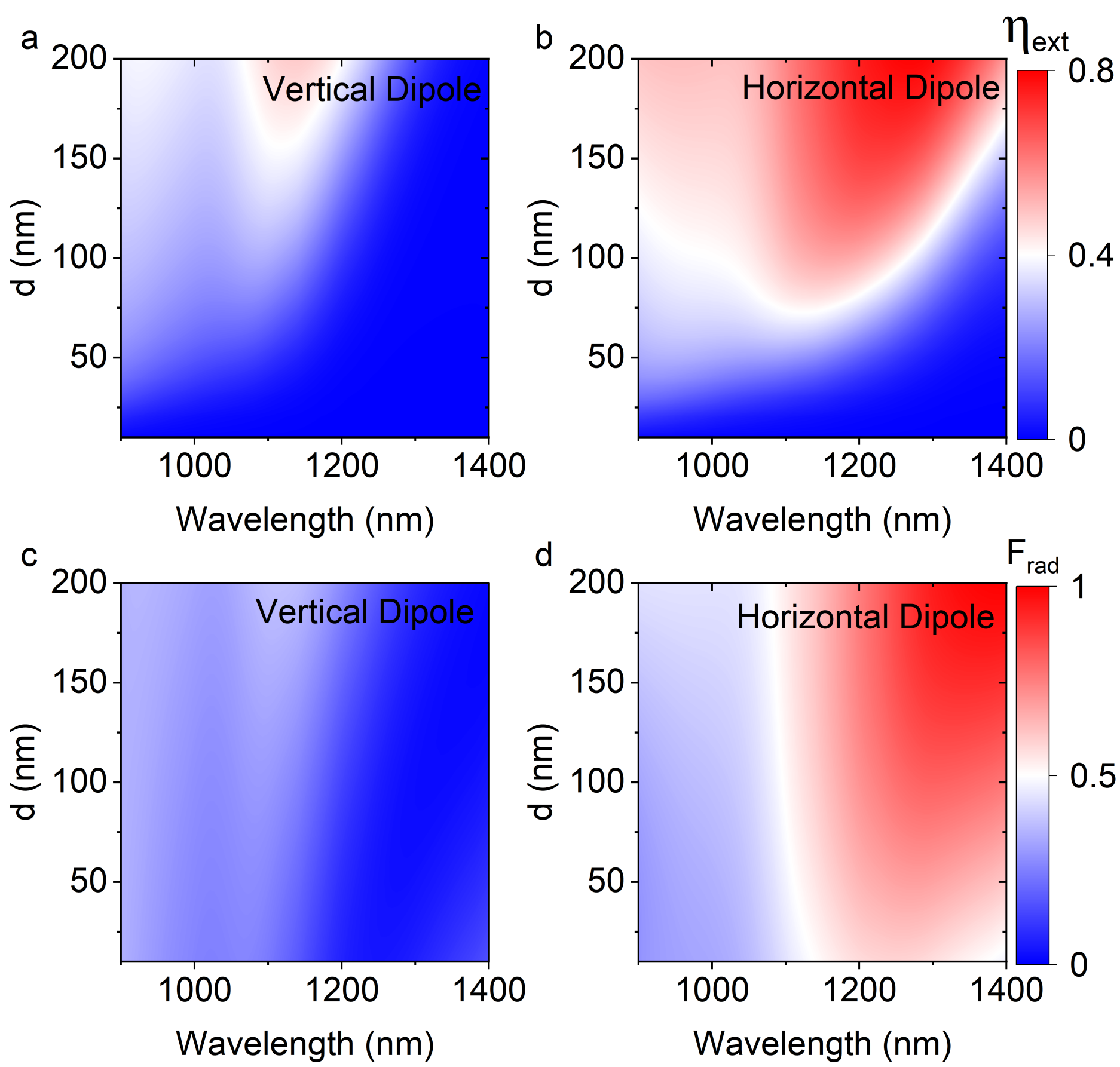

Figure S8: η_{ext} and F_{rad} on ITO substrate as a function of wavelength and dipole distance from substrate. (a): η_{ext} for vertical dipole, (b): η_{ext} for horizontal dipole, (c): F_{rad} for vertical dipole, and (d): F_{rad} for horizontal dipole.

Supplementary Figure S9: - F_{rad} for Isotropic dipole on Ag

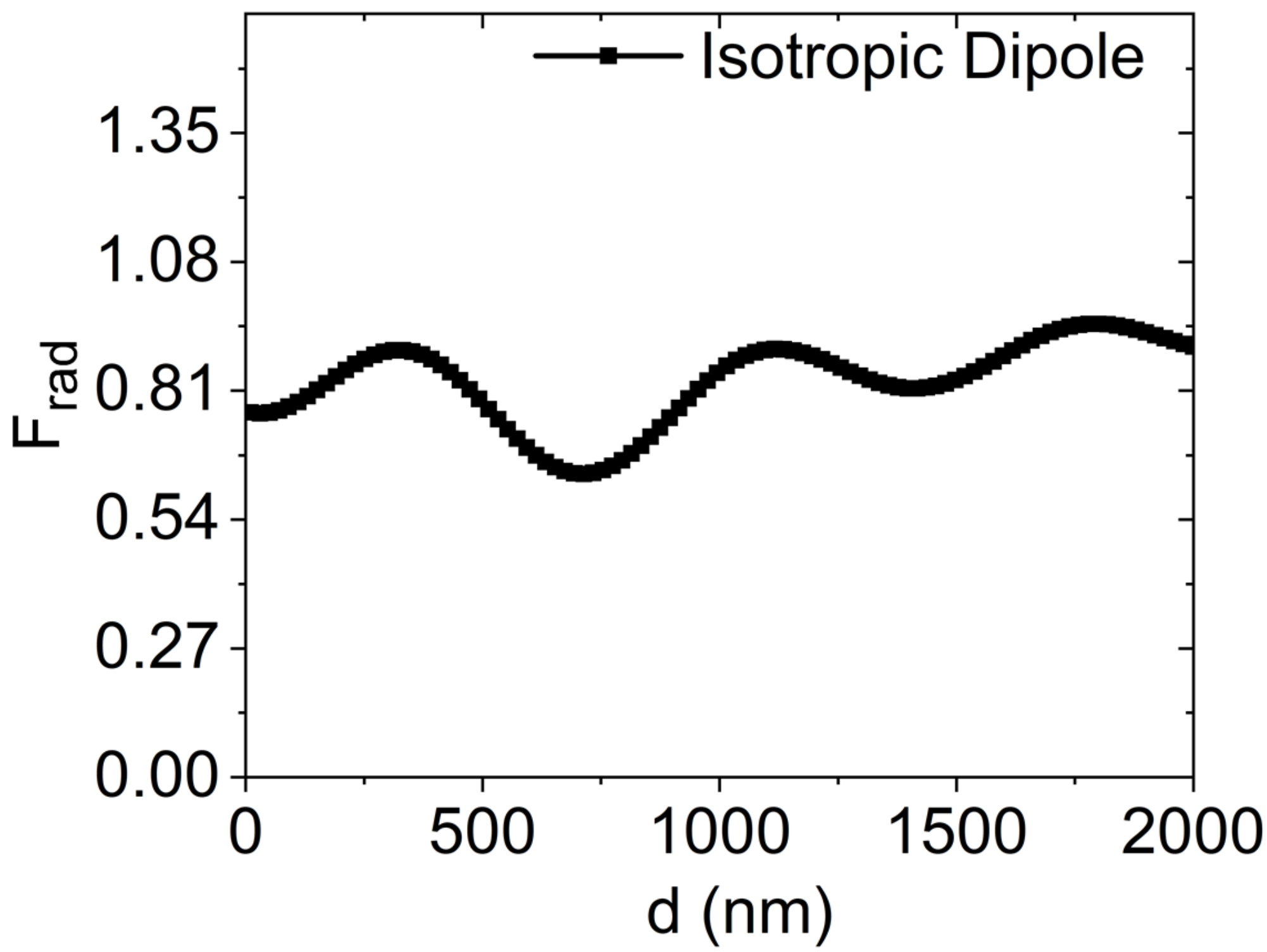

Figure S9: F_{rad} as a function of d for isotropic dipole near Ag substrate at a 1324 nm wavelength. The isotropic dipole consistently shows F_{rad} values less than 1 for all dipole distance d.

Supplementary Figure S10: - F_{rad} for Isotropic dipoles on ITO

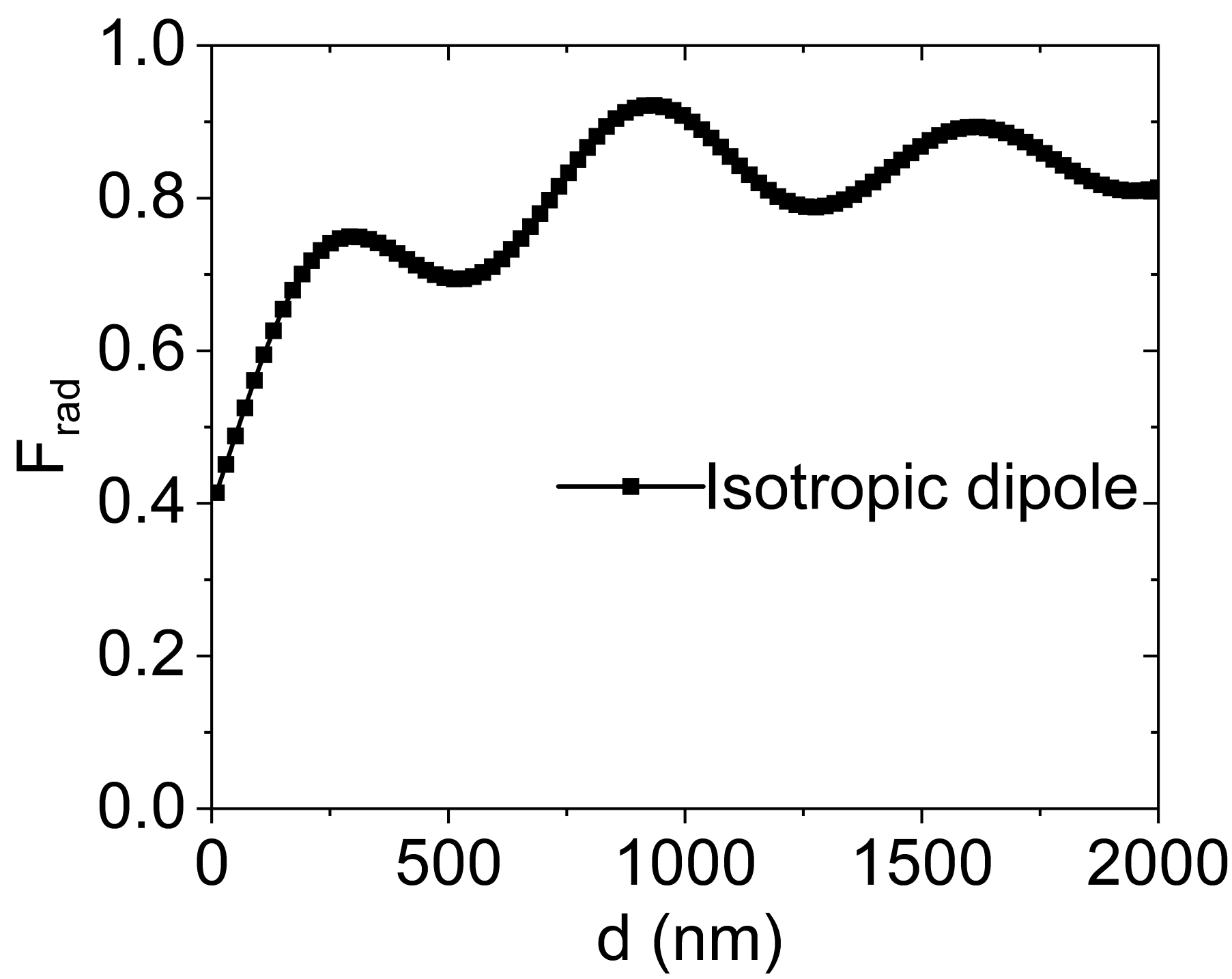

Figure S10: F_{rad} as a function of d for isotropic dipole near ITO substrate at a 1324 nm wavelength for isotropic dipoles.

Supplementary Figure S11: - Horizontal dipoles on dielectric with refractive index 4

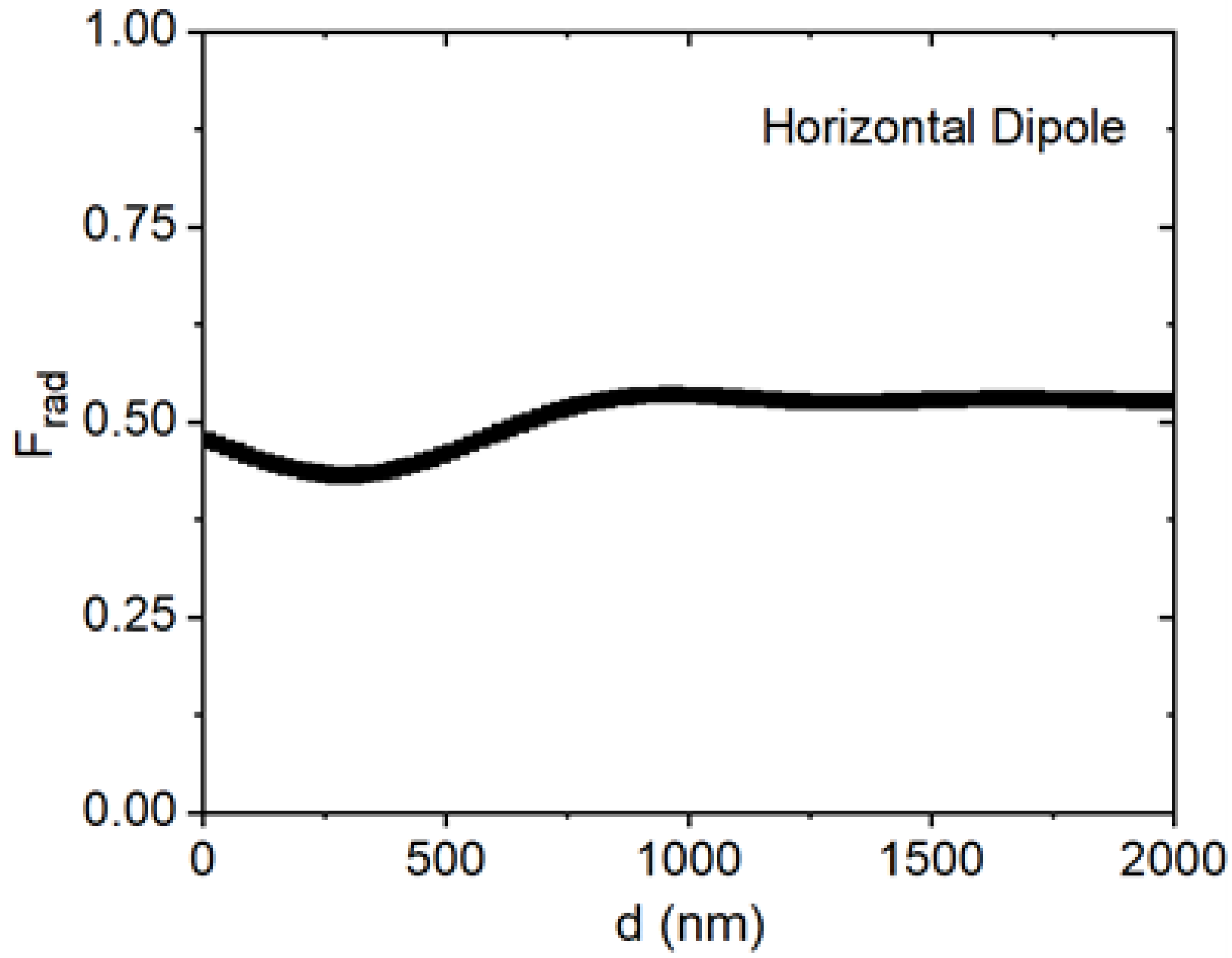

Figure S11: F_{rad} as a function of *d* for isotropic dipole near the dielectric with refractive index 4 at a 1324 nm wavelength for horizontal dipoles.

Supplementary Figure S12: - Comparing PF and F_{rad} using multilayer (ML) system and effective medium (EM) theory for ENZ MM

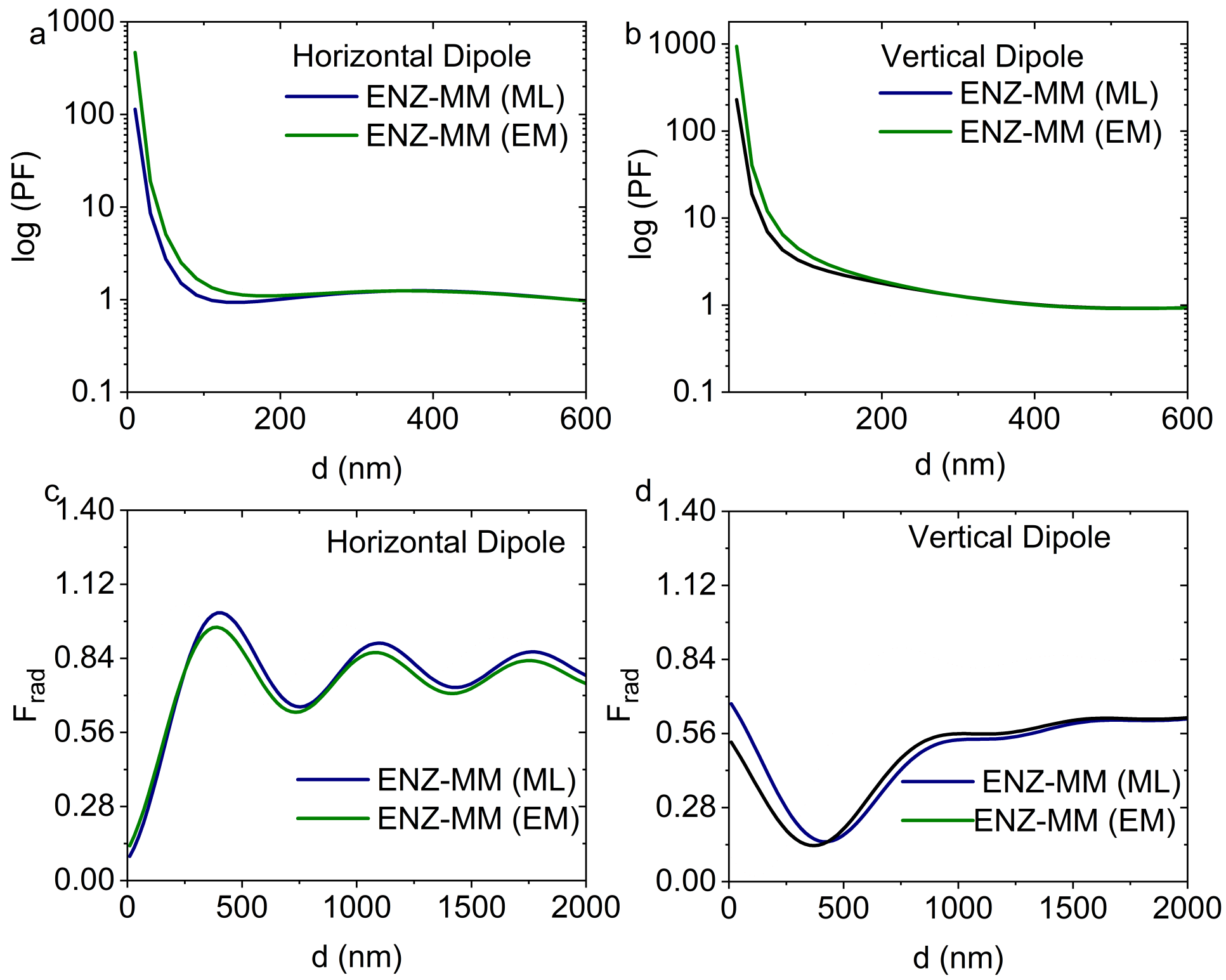

Figure S12: PF and F_{rad} as a function of *d* for horizontal and vertical dipole near ENZ-MM using ML and EM.

Supplementary Figure S13: Energy conservation and angular power balance

Figure S13 shows the calculated reflection (R), transmission (T), and absorption (A) as a function of the normalized in-plane wavevector k_x/k_0 at $\lambda = 1324$ nm for both p- and s-polarized light in the ENZ systems discussed in the main text. This representation directly captures the full angular spectrum relevant to dipole emission near an interface.

Figure S13(a) corresponds to the ideal lossless ENZ substrate. The response is dominated by nearly perfect reflection ($R \approx 1$) over most of the angular range for both polarizations, with negligible absorption. A narrow feature is observed for p-polarized light near normal incidence ($k_x/k_0 \approx 0$), where transmission exhibits a sharp peak associated with impedance matching at the interface. The narrow p-polarized transmission feature near $k_x/k_0 \approx 0$ is confined to a very small angular range ($\theta \lesssim 0.6°$), corresponding to $\Delta k_x/k_0 \sim 10^{-2}$. Although the transmission reaches a finite value at this point, its integrated contribution over k-space is negligible due to its extremely limited width. As a result, the overall response remains dominated by reflection ($R \approx 1$).Outside this narrow region, transmission remains negligible and reflection dominates.

Figures S13(b) and S13(c) show the corresponding results for ITO and the ENZ multilayer metamaterial (ENZ-MM), respectively. In these realistic systems, reflection, transmission, and absorption exhibit a strong angular dependence due to material losses and dispersion. In particular, absorption becomes significant and transmission is no longer negligible, especially near the light line, indicating that the ideal ENZ assumption ($R \approx 1$) does not generally hold.

Importantly, in all cases the condition $R + T + A = 1$ is satisfied across the full wavevector range, explicitly confirming energy conservation. These results provide a quantitative assessment of how electromagnetic energy is distributed among reflection, transmission, and absorption channels as a function of angle, and clearly delineate the regime in which the ENZ approximation is valid.

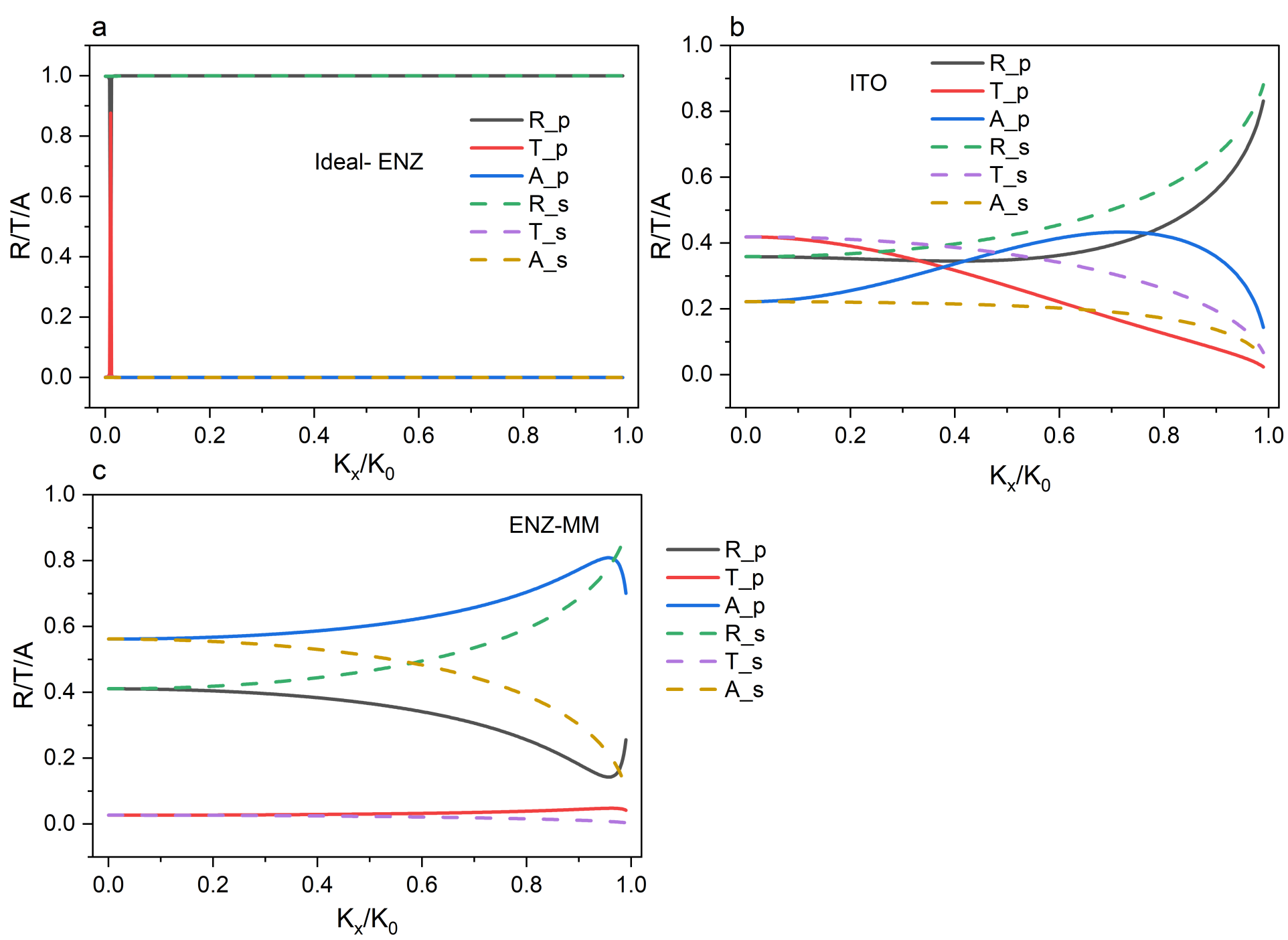

Figure S13 | Reflection (R), transmission (T), and absorption (A) as a function of normalized in-plane wavevector k_x/k_0 at $\lambda = 1324$ nm for both p- and s-polarized light. (a) Ideal lossless ENZ substrate, (b) ITO, and (c) ENZ multilayer metamaterial (ENZ-MM). The ideal ENZ case shows nearly perfect reflection over the full angular range with a dip in the p-polarized response near normal incidence, while ITO and ENZ-MM exhibit angle-dependent response due to material losses and dispersion.

Next, we compute the projected dissipated power spectra $dP_\perp/dk_x$ and $dP_\parallel/dk_x$, which are proportional to the local density of optical states (LDOS), in order to resolve the decay channels in k-space. This decomposition allows us to distinguish between propagating ($k_x/k_0 < 1$) radiative contributions and high-k ($k_x/k_0 > 1$) evanescent and non-radiative channels as a function of emitter–surface distance d.

Figure S14 shows the resulting k-space-resolved dissipated power for both perpendicular and parallel dipole orientations. Panels (a–c) correspond to the perpendicular dipole ($dP_\perp/dk_x$), while panels (d–f) correspond to the parallel dipole ($dP_\parallel/dk_x$), for the ideal ENZ, ITO, and ENZ multilayer systems, respectively. In the ideal ENZ case, the dissipated power is strongly confined to the low-k region ($k_x/k_0 < 1.5$), with negligible contributions from high-k modes for both dipole orientations. This indicates that emission is almost entirely into propagating channels, which explains why the total Purcell factor closely matches the radiative component in this limit. Integrating the spectrum over the high-k region ($k_x/k_0 > 1.5$) yields a vanishing contribution to the total dissipated power (within numerical precision, $\sim 0.00\%$), confirming that high-k channels are negligible in this case.

The weak signal visible in the logarithmic-scale plot therefore arises from low-k radiative modes coupled into the glass substrate ($n = 1.5$), rather than from genuine evanescent contributions.

In contrast, for ITO, the dissipated power exhibits a broad distribution extending well into the high-k region, particularly at small emitter–surface distances. This reflects strong coupling to evanescent and absorptive modes, which contribute to non-radiative decay channels. The ENZ multilayer metamaterial (ENZ-MM) shows even more pronounced high-k contributions, consistent with hyperbolic-type dispersion and an enhanced density of optical states at large in-plane wavevectors. This behavior leads to substantial deviations between the total decay rate and the radiative component, especially in the near-field regime.

Overall, the comparison between perpendicular and parallel dipoles highlights the expected polarization dependence of the LDOS, with parallel dipoles coupling more efficiently to high-k channels in ITO and ENZ-MM. These results explicitly demonstrate the transition from predominantly radiative emission in the ideal ENZ limit to strongly non-radiative and high-k-dominated behavior in realistic lossy systems.

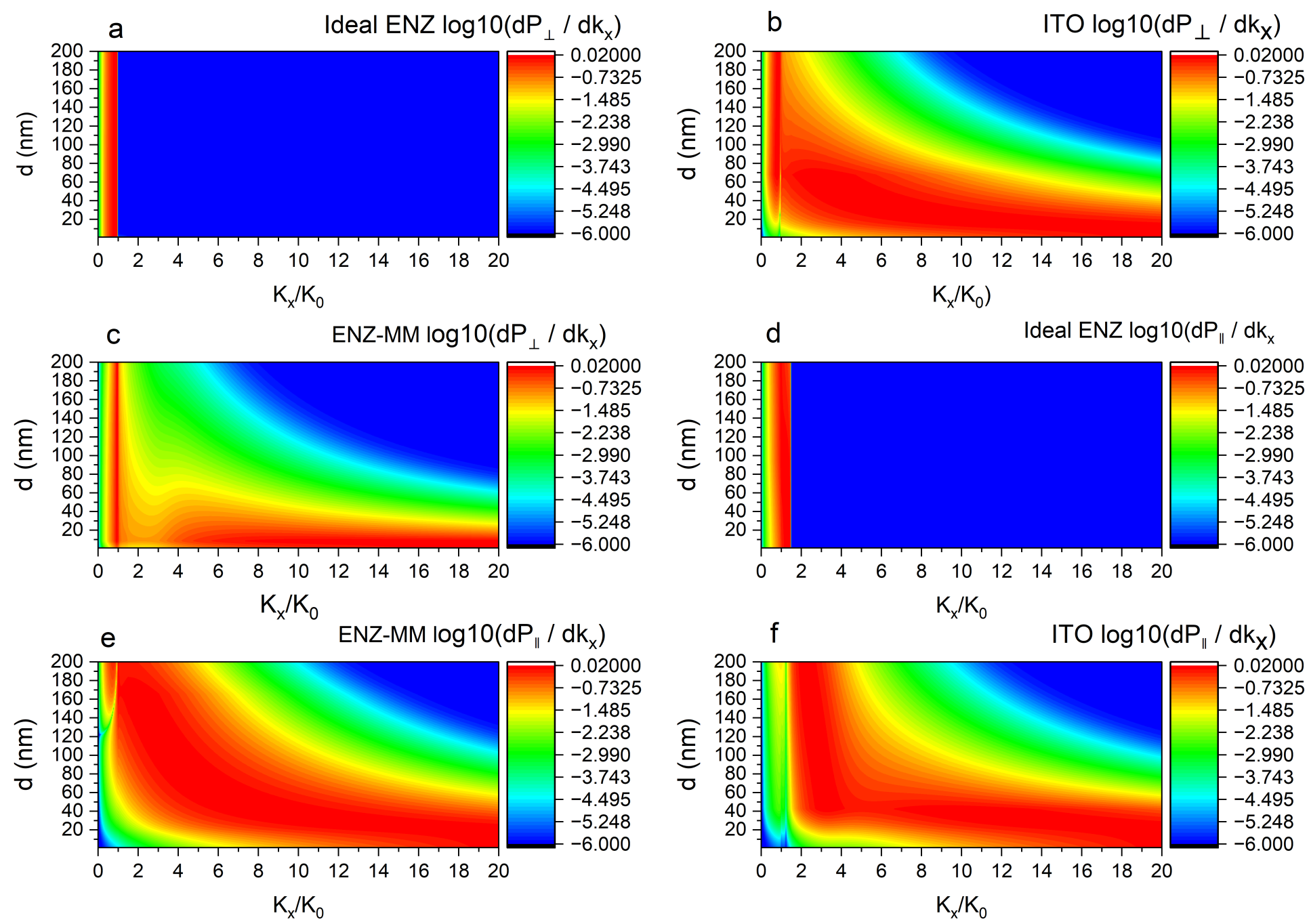

Figure S14 | Spectral decomposition of the projected dissipated power $dP_{\perp}/dk_x$ and $dP_{\|}/dk_x$ (LDOS) as a function of in-plane wavevector k_x/k_0 and emitter–surface distance d at $\lambda = 1324$ nm. Panels (a–c) show the perpendicular dipole response and panels (d–f) the parallel dipole response for (a,d) ideal ENZ, (b,e) ITO, and (c,f) ENZ multilayer (ENZ-MM). The ideal ENZ case is confined to low-k modes, while ITO and ENZ-MM exhibit strong high-k contributions due to lossy and evanescent channels, with ENZ-MM showing a hyperbolic-type response.

Supplementary Figure S15: Robustness of the ENZ-Induced Emission Behavior

To assess the robustness of the inverse Drexhage effect and the generality of our conclusions, we performed a systematic parameter sensitivity analysis, including variations in the real and imaginary parts of the permittivity, carrier concentration, multilayer structural parameters, and the validity of the effective medium approximation (EMA).

(a) Dependence on $\mathrm{Re}(\varepsilon)$**:** Figure S15(a) shows the radiative enhancement factor F_{rad} as a function of $\mathrm{Re}(\varepsilon)$ in the range -0.1 to 0.1, at a fixed $\mathrm{Im}(\varepsilon) = 0.001$ and dipole distance $d = 10$ nm. A pronounced inversion behavior is observed near $\mathrm{Re}(\varepsilon) \approx 0$, confirming that the inverse Drexhage effect is tightly linked to the ENZ condition. The horizontal dipole exhibits a peak at $\mathrm{Re}(\varepsilon) = 0$, while the vertical dipole shows a minimum, consistent with the phase-driven interference mechanism discussed in the main text. This sweep defines the finite spectral window over which the ENZ response is effective.

(b) Carrier concentration sensitivity (ITO): Figure S15(b) presents F_{rad} for both dipole orientations as a function of carrier concentration in ITO, varied by $\pm 10\%$ around the nominal value. The results show only modest variation in F_{rad}, indicating that the emission behavior is robust against realistic fabrication tolerances in carrier density.

(c) Multilayer structural dependence (ENZ-MM): Figure S15(c) shows the effect of varying the metal fill fraction in the ENZ multilayer (while keeping the total period constant). The radiative enhancement remains qualitatively unchanged across a wide range of fill fractions, demonstrating that the observed behavior is not the result of fine-tuned geometry but persists across realistic structural variations.

(d) Validity of effective medium approximation : Figure S15(d) quantifies the relative deviation between the multilayer (ML) calculation and the effective medium (EM) approximation, defined as $|F_{\mathrm{rad,ML}} - F_{\mathrm{rad,EM}}|/F_{\mathrm{rad,ML}}$, plotted on a logarithmic

scale as a function of dipole distance d. The results show that the deviation increases in the near-field regime due to the breakdown of the effective medium approximation at small emitter–surface separations. Using a 10% error threshold, the effective medium description remains quantitatively valid down to approximately $d \sim$ 10–15 nm for the parallel dipole and $d \sim$ 20–30 nm for the perpendicular dipole. The difference between the two orientations arises from their distinct coupling to high-k modes, with the parallel dipole exhibiting stronger sensitivity to near-field and evanescent contributions. At larger distances, the deviation decreases and remains well below 10%, confirming the validity of the effective medium approximation for describing the far-field emission behavior.

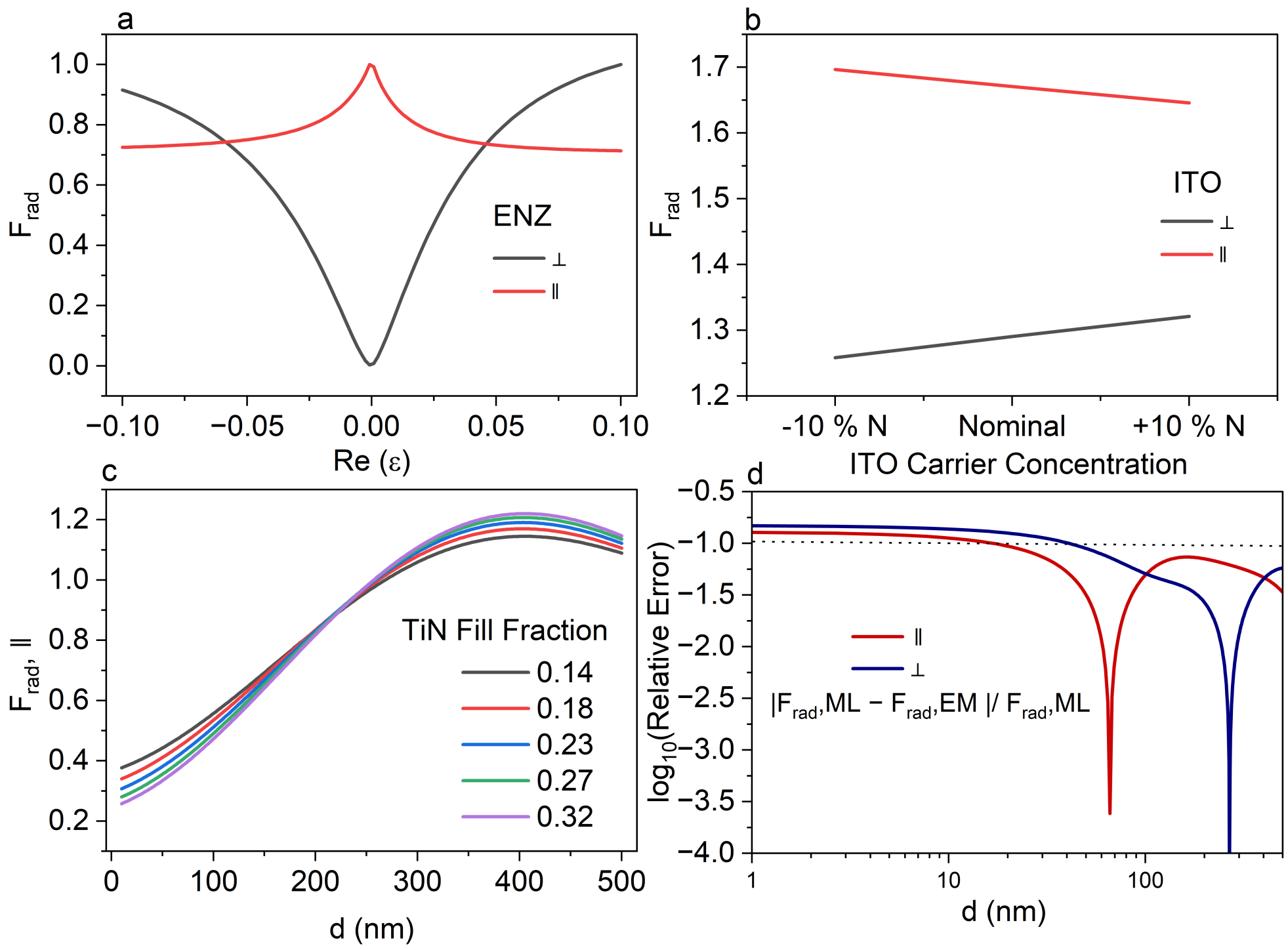

Figure S15 | Robustness analysis of the ENZ-induced inverse Drexhage effect. (a) Dependence of F_{rad} on $\text{Re}(\varepsilon)$ near the ENZ condition. (b) Sensitivity to ITO carrier concentration variations. (c) Dependence on the metal fill fraction in the ENZ multilayer. (d) Relative deviation between multilayer and effective medium calculations as a function of dipole distance d.

[1] D. Lu, J. J. Kan, E. E. Fullerton, and Z. Liu, "Enhancing spontaneous emission rates of molecules using nanopatterned multilayer hyperbolic metamaterials," Nat. Nanotechnol. **9**, 48–53 (2014).

[2] S. K. Chamoli, M. ElKabbash, J. Zhang, and C. Guo, "Dynamic control of spontaneous emission rate using tunable hyperbolic metamaterials," Opt. Lett. **45**, 1671–1674 (2020).
